# Metal–organic B←N frameworks

**DOI:** 10.1039/d6sc04671a

**Published:** 2026-08-06

**Authors:** Atena B. Solea, Sophia Outenah, Farzaneh Fadaei-Tirani, Kay Severin

**Affiliations:** a Institut des Sciences et Ingénierie Chimiques, École Polytechnique Fédérale de Lausanne (EPFL) 1015 Lausanne Switzerland kay.severin@epfl.ch

## Abstract

Metal–organic B←N frameworks containing gold complexes were obtained by linking Au_3_(pyrazolate)_3_ complexes to di- or triboronate esters *via* dative boron–nitrogen bonds. The dynamic nature of the B←N interactions facilitated the formation of highly crystalline materials, enabling structural characterization by single-crystal X-ray diffraction. Both the one- and two-dimensional polymers showed substantial solvent-filled voids. Solution-phase exchange of the solvents with halobenzenes induced pronounced structural changes of the polymeric frameworks. The combination of crystallinity and structural adaptability demonstrates the potential of dative B←N bonds in the design of new metal-based framework materials.

## Abstract 2

Crystalline polymers containing trinuclear gold complexes are accessible by linking Au complexes to polyboronate esters *via* dative boron–nitrogen bonds. The polymers are porous and structural changes are observed upon exchange of guest molecules.

## Graphical abstract

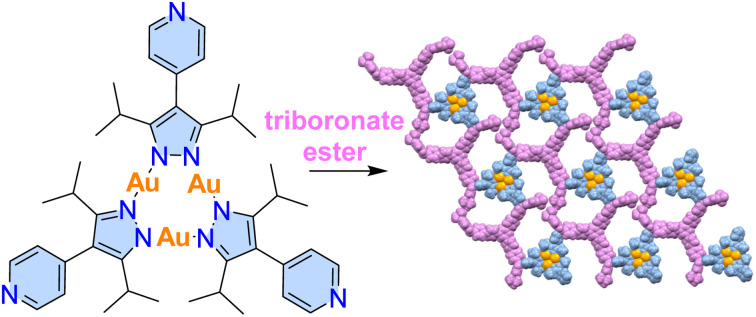

## Introduction

**Fig. 1:**
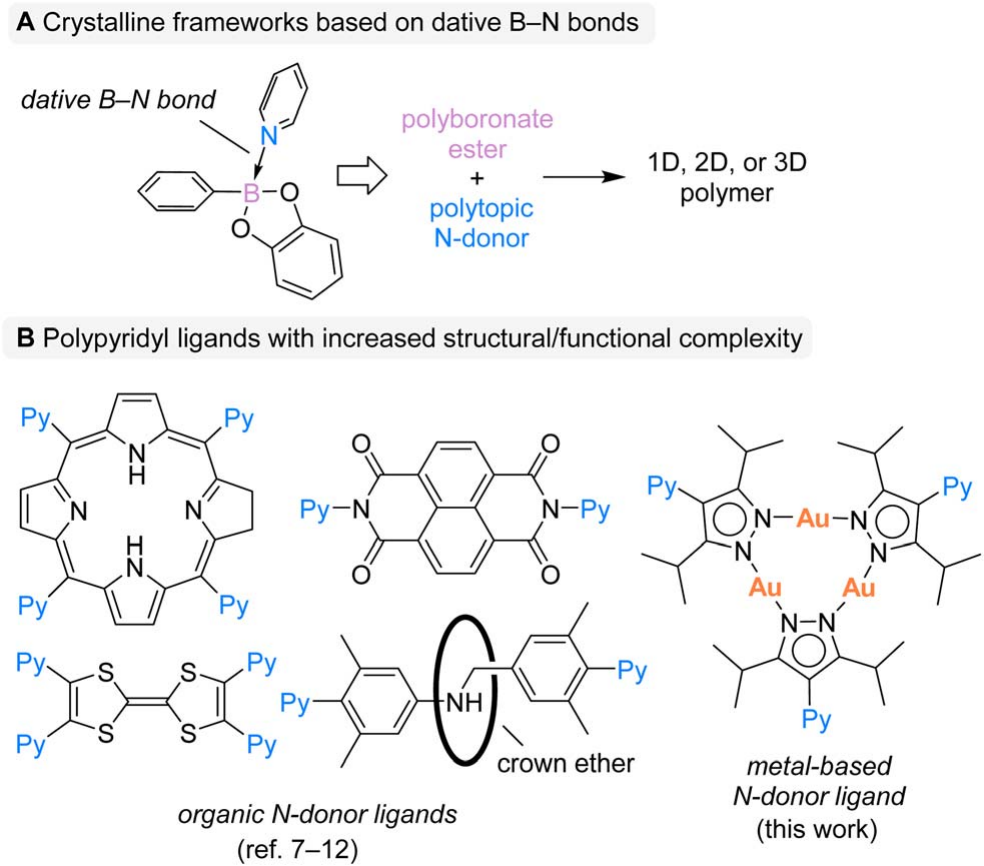
(A) Polymeric materials based on dative B←N bonds can be obtained by combining polyboronate esters with polytopic N-donor ligands. (B) The structural and/or functional complexity of B←N frameworks can be increased by using special organic N-donor ligands, or, as described herein, by using a metal-based N-donor ligand.

Boronate esters are capable of forming Lewis acid–base adducts with N-donor ligands such as pyridines. This reversible dative interaction can be leveraged to construct framework materials by combining polyboronate esters with polytopic N-donor linkers (Fig. 1A).^1,2^ The feasibility of this synthetic approach was first demonstrated in 2011. Crystalline two-dimensional B←N networks were prepared from a tritopic boronate ester and dipyridyl ligands.^3^ Since then, the scope of this chemistry has expanded considerably, and a variety of one-, two-, and three-dimensional B←N polymers have been prepared from polyboronate esters and polytopic N-donor ligands.^1,2^ In addition to boronate esters, boroxines have been used as the Lewis-acidic component.^1,2^

A notable feature of these B←N networks is the possibility to obtain highly crystalline materials, which can be analyzed by single-crystal X-ray diffraction (XRD). The high degree of crystallinity represents an advantage over other materials based on light elements, such as covalent organic frameworks (COFs). For the latter, obtaining single crystals represent a challenging task.^4^

The first B←N networks were constructed using relatively simple polypyridyl ligands such as 4,4′-bipyridyl,^3,5,6^ 1,2-di(4-pyridyl)ethylene,^3,5^ or tri(4-pyridyl)-1,3,5-triazine.^5^ With the aim of creating functionally and/or structurally more complex B←N polymers, more elaborate polypyridyl ligands were employed in recent years (Fig. 1B). For example, crystalline polymers were obtained with polypyridyl ligands having porphyrin,^7,8^ naphthalenetetracarboxdiimide,^9–11^ or tetrathiafulvalene spacers,^7^ and a rotaxane-based dipyridyl ligand gave rise to polyrotaxane crystals.^12^ These materials have been explored for applications such as photocatalytic H_2_O_2_ production,^8^ energy storage,^9^ optoelectronic devices,^7^ and the construction of elastic crystals.^12^

Beyond purely organic building blocks, metal complexes with reactive side chains have been used with great success for the synthesis of COF-like materials, sometimes referred to as ‘covalent metal–organic frameworks’.^13,14^ The analogous use of metal-based N-donor ligands for the formation of B←N networks has not been reported.

In order to construct metal–organic B←N networks, we turned to Au_3_(pyrazolate)_3_ complexes as potential building blocks. These complexes constitute an important class of coordination compounds in inorganic and materials chemistry.^15^ They display variable π-basicity/acidity, which can be modulated by the substituents on the pyrazolate ligand.^16,17^ Aurophilic interactions promote the self-assembly of Au_3_(pyrazolate)_3_ complexes, and the resulting aggregates are often highly luminescent.^15–19^ Additional attractive features of these complexes include facile synthetic access and the ability to decorate them with a wide range of functional groups.^20–23^ Of particular importance for the present work is the inert character of Au_3_(pyrazolate)_3_ complexes.^24^ The resistance to ligand exchange reactions was expected to enhance their compatibility with B←N bond formation.

Below, we describe the first examples of metal–organic B←N frameworks. Specifically, we report the synthesis, the structural characterisation, and the adaptive behavior of one- and two-dimensional B←N polymers featuring Au_3_(pyrazolate)_3_ complexes as connecting nodes (Fig. 1B).

## Results and discussion

**Scheme 1:**
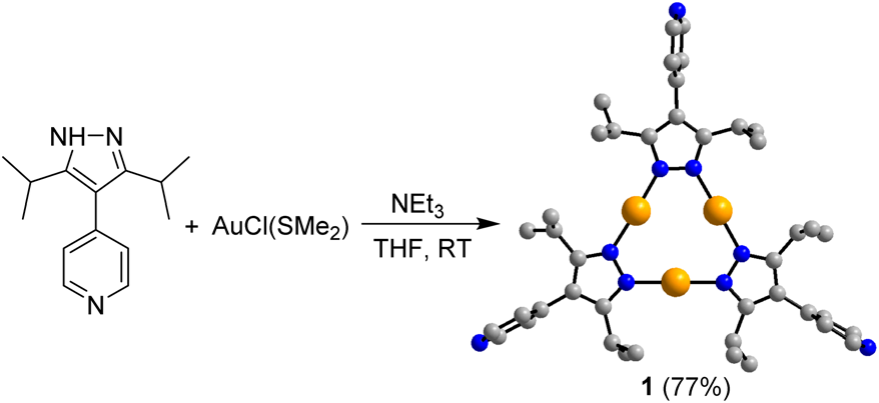
Synthesis of complex 1. The structure of the product is based on a crystallographic analysis. Hydrogen atoms are not shown.

For our investigations, we prepared the Au^I^ complex 1 (Scheme 1). It features three divergent 4-pyridyl substituents as N-donor groups. Moreover, the bridging pyrazole ligands carry isopropyl groups, which were expected to enhance the solubility of 1 in organic solvents.

Complex 1 was obtained in 77% yield by the reaction of 4-[3,5-bis(propan-2-yl)-1*H*-pyrazol-4-yl]pyridine^25^ with AuCl(SMe_2_) in the presence of Et_3_N. The trinuclear structure of complex 1 was corroborated by NMR spectroscopy, high-resolution mass spectrometry, and single-crystal XRD. Intermolecular aurophilic interactions in the solid state were not observed.

**Scheme 2:**
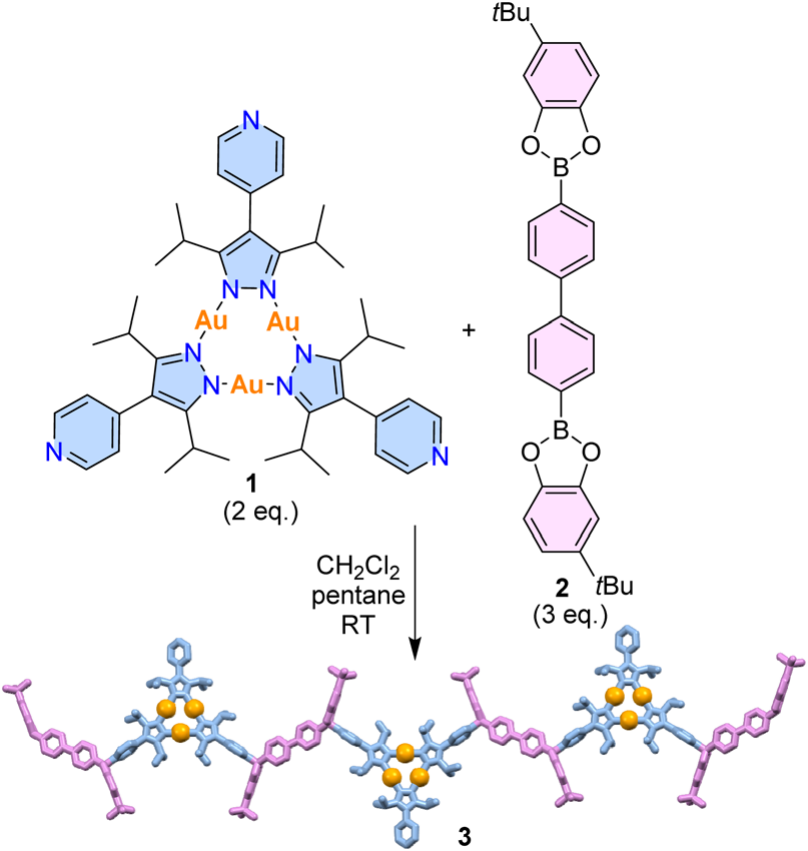
Synthesis of polymer 3. The structure of the product is based on a crystallographic analysis. Hydrogen atoms are not shown.

With the aim of generating a (2,3)-connected two-dimensional B←N network, we prepared a solution of complex 1 (2 equiv.) and the diboronate ester 2 (3 equiv.) in dichloromethane. Slow vapor diffusion of pentane into the dichloromethane solution resulted in the formation of yellow crystals, which were analyzed by single-crystal XRD. The analysis revealed that a one-dimensional polymer (3) had formed (Scheme 2).

Polymer 3 shows a ‘zigzag’ geometry, with a *trans* configuration of the dioxaborole groups. Two of the three pyridyl groups of the Au complexes are involved in dative B←N bonds. The latter have a length of 1.665(16) Å, which is in the range found for pyridine-dioxaborole adducts.^1,2^ The third pyridyl group of the Au trimers in polymer 3 is not coordinated to a boronate ester, despite the fact that we had used a 2 : 3 ratio of the building blocks. Such behavior is not unusual: the formation of one-dimensional B←N polymers from tri- and tetratopic N-donor ligands has been reported before.^5,7^

A notable feature of crystalline 3 is the large void space between the polymer chains. The solvent-accessible volume represents 37% of the unit cell volume. Residual electron density suggests that the voids in 3 are occupied by six disordered dichloromethane molecules for each Au trimer. However, the highly disordered solvent molecules could not be modelled explicitly, and their contribution to the residual electron density was therefore treated using the solvent-mask procedure implemented in Olex2.^26^

**Scheme 3:**
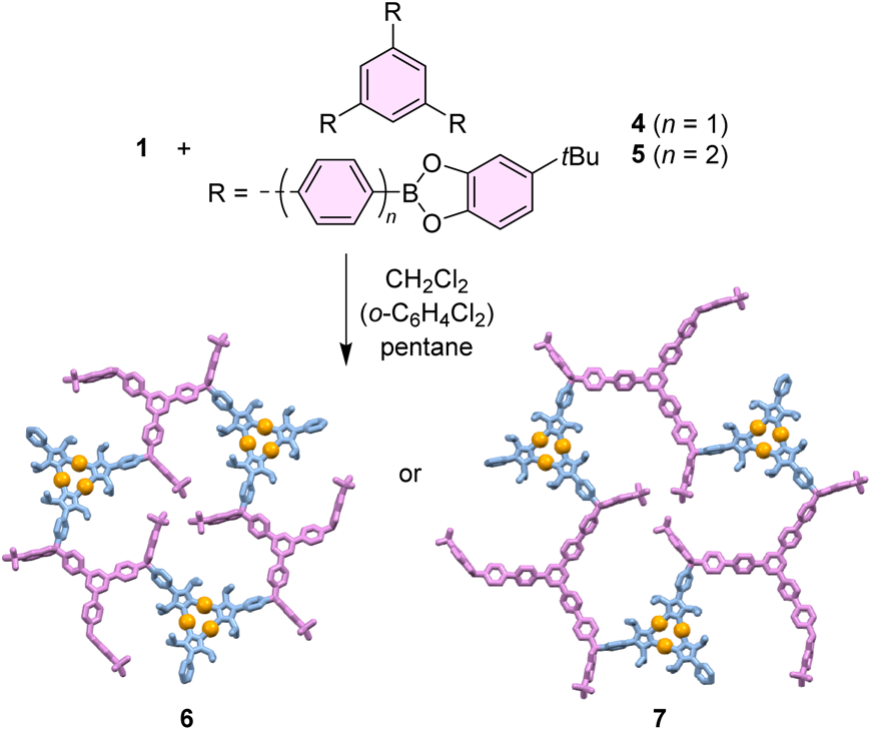
Synthesis of the two-dimensional networks 6 and 7. The graphic representations of the products are based on crystallographic analyses. The graphics show the macrocyclic subunits of the two-dimensional polymers. Hydrogen atoms are not shown.

Next, we examined the crosslinking of complex 1 with triboronate esters. Two different esters were employed, 4 and 5,^3^ both of which display *C*_3_ symmetry (Scheme 3). An equal amount of complex 1 and the respective ester was mixed in dichloromethane or a mixture of dichloromethane and *ortho*-dichlorobenzene, and pentane was used to induce crystallization. This procedure resulted in the formation of the two-dimensional crystalline networks 6 and 7 (Scheme 3).

**Fig. 2:**
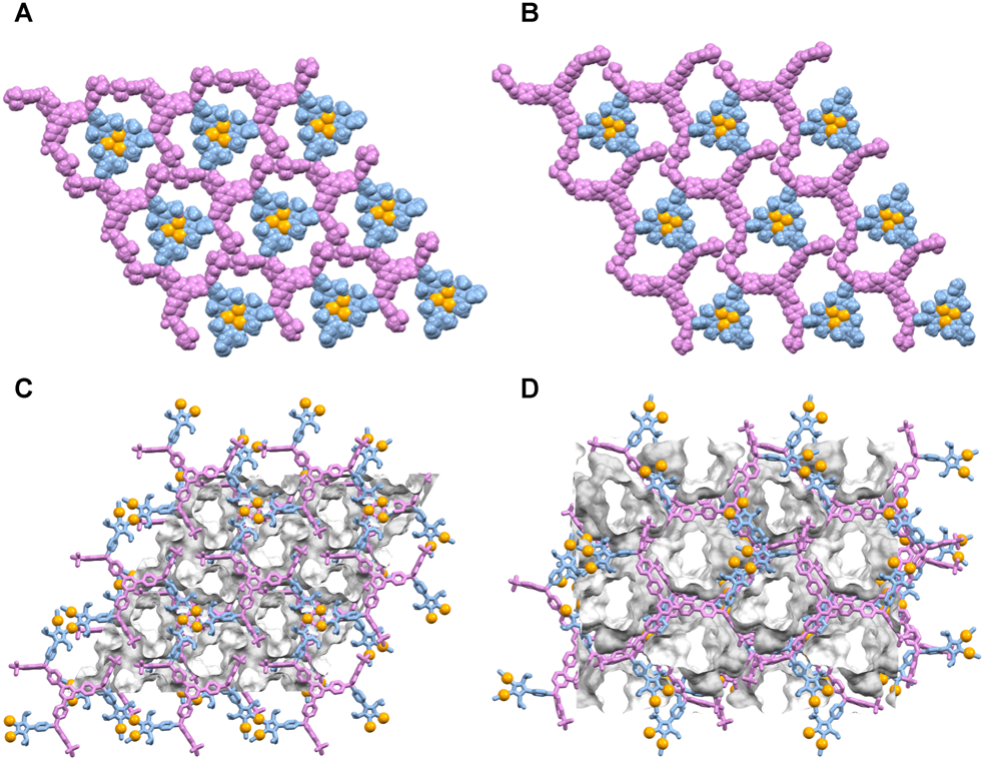
(A) Structure of the two-dimensional network 6. (B) Structure of the two-dimensional network 7. (C) Visualization of the pore structure in 6. (D) Visualization of the pore structure in 7.

Crystallographic analyses of the polymers revealed similar overall structures. Crosslinking *via* dative B←N bonds had resulted in the formation of two-dimensional, (3,3)-connected networks (Fig. 2A and B). Thus far, only a limited number of two-dimensional B←N networks have been structurally characterized. The reported examples have (2,3)-connected^3,27^ or (2,4)-connected network structures.^5,6,8,28^ The (3,3)-connectivity observed for 6 and 7, is, to the best of our knowledge, unprecedented.

The layers in network 6 show an (A–B–C)_*n*_ stacking sequence, with an average interlayer distance of 5.5 Å. Each layer is composed of 84-membered macrocycles, which are made from three Au CTS and three triboronate esters (Scheme 3). The relative position of the layers allows for close contacts between the Au complexes in one layer and the aromatic core of the triboronate esters in an adjacent layer, with a centroid–centroid distance of 3.62 Å. Attractive interactions between Au_3_(pyrazolate)_3_ complexes and aromatic compounds are well documented,^15,16,29^ and the presence of these interactions in 6 is expected to stabilize the overall structure. The B←N bonds in 6 are rather short (1.582(14) Å), indicating strong dative bonds. The three-dimensional structure of 6 shows channels along the crystallographic *c*-axis with a diameter of around 5.7 Å (Fig. 2C). The total void volume in 6 represents 42% of the volume of the unit cell, and the calculated surface area is 557 m^2^ g^−1^.

The layers in network 7 have an (A–B)_*n*_ repeat pattern, with an interlayer distance of 4.3 Å. Due to the additional phenylene spacers in the triboronate ester 5, the macrocycles in 7 are larger, with a ring size of 108 atoms. Consequently, larger pores are observed, with a diameter of around 8.3 Å (Fig. 2D). The calculated surface area of 1171 m^2^ g^−1^ is approximately twice that found for network 6. Close contacts between the Au complexes and the aromatic core of the triboronate esters, as observed for 6, are not found for network 7. The lengths of the B←N bonds in 7 are 1.648(19), 1.66(2), and 1.69(2) Å. These values are larger than what was found for 6, but still within the range observed for B←N networks.^1,2^

Both networks display good thermal stability, as shown by thermogravimetric analyses (see the SI, Fig. S8). Moreover, the materials remain intact upon exposure to water, as demonstrated by FT-IR spectra recorded before and after soaking the samples in water for 16 h (see the SI, Fig. S7).

Since the polymers 3, 6, and 7 all show substantial voids in their crystal lattice, we have examined whether the materials would display permanent porosity. For 3 and 7, it was challenging to obtain larger quantities of single-crystalline material, and as-synthesized polymers were used for BET measurements. For network 6, on the other hand, single-crystalline samples were employed. BET measurements revealed low apparent surface areas for all samples (<50 m^2^ g^−1^), indicating structural collapse upon thermal activation under vacuum. In line with this hypothesis, the powder XRD data of sample 6 after activation indicated the presence of largely amorphous material (see the SI, Fig. S13). The lack of permanent porosity is not unusual for B←N networks: only a few examples of networks with apparent surface areas larger than 100 m^2^ g^−1^ have been reported thus far.^6,27,28,30^

**Fig. 3:**
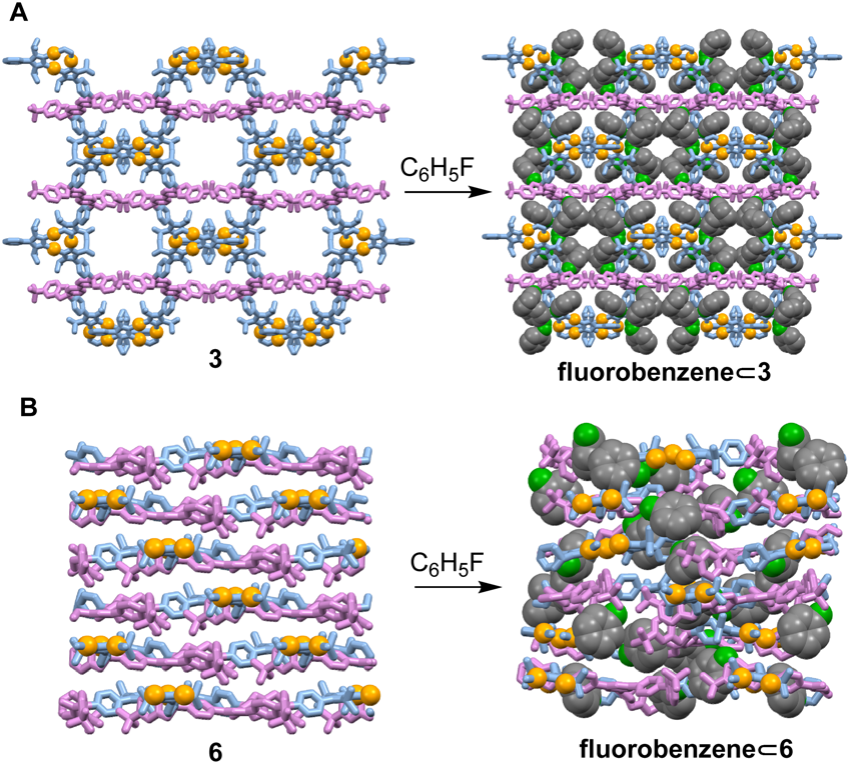
(A) Graphic representations of the structure of polymers 3 before and after exposure to fluorobenzene. Hydrogen atoms and solvent molecules in 3 are not shown. (B) Graphic representations of the structure of network 6 before and after exposure to fluorobenzene. Hydrogen atoms and solvent molecules in 6 are not shown.

In addition to BET measurements, we examined whether the new B←N polymers would allow for solution-phase exchange reactions of guest molecules. Single crystals of 3, 6, and 7 were soaked for 24 h in fluorobenzene. Subsequently, XRD analyses were performed. For the one-dimensional polymer 3, we observed the presence of six fluorobenzene molecules for each Au trimer (Fig. 3A). The incorporation of fluorobenzene led to an 8.9% increase in the unit cell volume, primarily due to expansion along the crystallographic *c* axis, which increased in length by 12% (from 30.1756(8) to 33.6992(4) Å).

For covalent organic frameworks (COFs), it is well established that a post-synthetic exchange of guest molecules can result in notable structural changes.^31^ Guest-induced structural reorganization of this magnitude is rare in B←N polymers, underscoring the unusual combination of crystallinity and adaptivity in the present system.^10,12,32^

Network 6 was also able to absorb fluorobenzene, as well as the structurally-related chlorobenzene and bromobenzene, as shown by XRD analyses after soaking (Fig. 3B, S10 and S11). Five and a half fluorobenzene molecules were found for each Au trimer. Out of these, two fully occupied and one half-occupied fluorobenzene molecules could be modelled crystallographically, while the remaining three were severely disordered, so a solvent mask was applied, as implemented in Olex2.^26^ Importantly, the incorporation of fluorobenzene in 6 led again to substantial changes in the network structure. The (A–B–C)_*n*_ repeat pattern was replaced by a six-layer repeat motif, and the average interlayer distances decreased from 5.5 to 5.0 Å. The layers in the fluorobenzene-containing network adopt a wave-like geometry, in contrast to the flatter geometry observed for the pristine structure. Moreover, the stacking of gold trimers over the aromatic rings of the triboronate esters was disrupted by the incorporation of fluorobenzene. Chlorobenzene and bromobenzene produced the same changes in unit cell parameters as observed for fluorobenzene: the (A–B–C)_n_ pattern was again replaced by a six-layer repeat motif, with unit cell parameters nearly identical to those of the fluorobenzene-soaked network (see the SI, Fig. S10 and S11). Six molecules of chlorobenzene or bromobenzene were found per Au trimer. For the chlorobenzene-soaked structure, all six could be modelled crystallographically; for bromobenzene, only two could be modelled, and the remainder were accounted for using a solvent mask, as implemented in Olex2.^26^

For network 7, the quality of the XRD data after soaking was unfortunately not sufficient to determine if and how much fluorobenzene was incorporated.

## Conclusions

The use of dative B←N bonds represents a versatile strategy for the construction of functional materials. To date, B←N frameworks have been derived exclusively from organic building blocks. We have now shown that metal complexes can be integrated into B←N polymers through the use of the functionalized Au_3_(pyrazolate)_3_ complex 1 as N-donor ligand. This approach afforded three metal–organic B←N frameworks: the one-dimensional polymer 3, which adopts a ‘zigzag’ structure with pendant pyridyl groups, and the two-dimensional networks 6 and 7, which feature an unprecedented (3,3)-connectivity. Halobenzene sorption experiments revealed that polymers 3 and 6 possess accessible porosity. The incorporation of guest molecules resulted in substantial structural reorganization of the frameworks.

It is worth noting that the polymers were obtained in highly crystalline form and were therefore amenable to single-crystal XRD analysis. In contrast, previous attempts to construct COF-like networks from Au_3_(pyrazolate)_3_ complexes yielded only microcrystalline materials with poor long-range order.^33,34^

Overall, our findings highlight the potential of metal-based building blocks in the construction of B←N networks and point toward new opportunities for the development of functional materials.

## Author contributions

A. B. S. and K. S. initiated the study, A. B. S. and S. O. performed the experiments and analyzed the data, F. F.-T. collected and processed the X-ray data, and A. B. S. and K. S. co-wrote the manuscript. All authors discussed the results and commented on the manuscript.

## Conflicts of interest

There are no conflicts to declare.

## Supplementary Material

SC-OLF-D6SC04671A-s001

SC-OLF-D6SC04671A-s002

## Data Availability

The data supporting this article have been included as part of the supplementary information (SI). Supplementary information: synthetic procedures and experimental details. See DOI: https://doi.org/10.1039/d6sc04671a. CCDC 2555662, 2555663, 2555664, 2555665, 2555666, 2555667, 2575900 and 2575901 contain the supplementary crystallographic data for this paper.^35*a*–*h*^
