# Supplementary material for: Metal–organic B←N frameworks
Source: Chem Sci. 2026 Aug 6;17(37):17764–8. doi: 10.1039/d6sc04671a (PMC13446452; doi:10.1039/d6sc04671a)
Supplement: Supplementary file 1 [file SC-017-D6SC04671A-s001.pdf]

Supplementary Information (SI) for Chemical Science.
This journal is © The Royal Society of Chemistry 2026

Supplementary Information

Metal-organic B←N frameworks

Atena B. Solea,[a] Sophia Outenah,[a] Farzaneh Fadaei-Tirani,[a] and Kay Severin*[a]

[a] *Institut of Chemical Sciences and Engineering, École Polytechnique Fédérale de Lausanne (EPFL), 1015 Lausanne, Switzerland*

Table of contents

1 General *3*

2 Synthetic procedures *3*

2.1 Synthesis of complex 1 .... **3**

2.2 Synthesis of compound 2 .... **5**

2.3 Synthesis of crystalline B–N coordination polymer 3 .... **7**

2.4 Synthesis of the crystalline 2D B–N network 6 .... **7**

2.5 Synthesis of the crystalline 2D B–N network 7 .... **7**

2.7 Synthesis and activation of amorphous B–N networks **7**

3. BET studies *8*

4. FT-IR of 6 and 7 before and after H_2O exposure *9*

5. TGA of amorphous powders of 6 and 7 .... *9*

6. Single-crystal X-ray diffraction *10*

6.1 Crystal data and experimental for 1 .... **10**

6.2 Crystal data and experimental for 3 .... **11**

6.3 Crystal data and experimental for fluorobenzene⊂3 .... **12**

6.4 Crystal data and experimental for 6 .... **14**

6.5 Crystal data and experimental for fluorobenzene⊂6 .... **15**

6.6 Crystal data and experimental for chlorobenzene⊂6 .... **16**

6.7 Crystal structure of bromobenzene⊂6 .... **18**

6.8 Crystal data and experimental for 7 .... **20**

7. Powder X-Ray Diffraction *22*

1 General

All chemicals were obtained from commercial sources and used without further purification unless stated otherwise. NMR spectra were measured on a Bruker Avance III HD spectrometer (^{1}H: 400 MHz) equipped with a BBFO-Plus$_z$ 5 mm probe or on a Bruker Avance III HD spectrometer (^{1}H: 600 MHz) equipped with a Prodigy BBFO 5 mm probe. High-resolution mass spectrometry was carried out using a hybrid ion trap-Orbitrap Fourier transform mass spectrometer, Orbitrap Elite (Thermo Scientific) equipped with a TriVersa Nanomate (Advion) nano-electrospray ionization source or with an Orbitrap Exploris 240 (Thermo Scientific) mass spectrometer equipped with a Sicrit (Plasmion) ionization source. FT-IR spectra were acquired on a Perkin-Elmer Spectrum-One instrument with diamond-anvil configuration. TGA measurements were performed on a TGA 4000 from Perkin Elmer, under N_2. Nitrogen (77 K) sorption measurements were performed on a Quantachrome Autosorb iQ analyzer. Powder diffraction measurements were performed on Rigaku Supernova diffractometer in powder diffraction mode. Data were measured between 3 and 53 ° 2 θ at 1 s per step of 0.01 and using a non-monochromated Cu-source. 4-(3,5-Diisopropyl-1H-pyrazol-4-yl)pyridine was prepared according to an adapted reported procedure.[1]

2 Synthetic procedures

2.1 Synthesis of complex 1

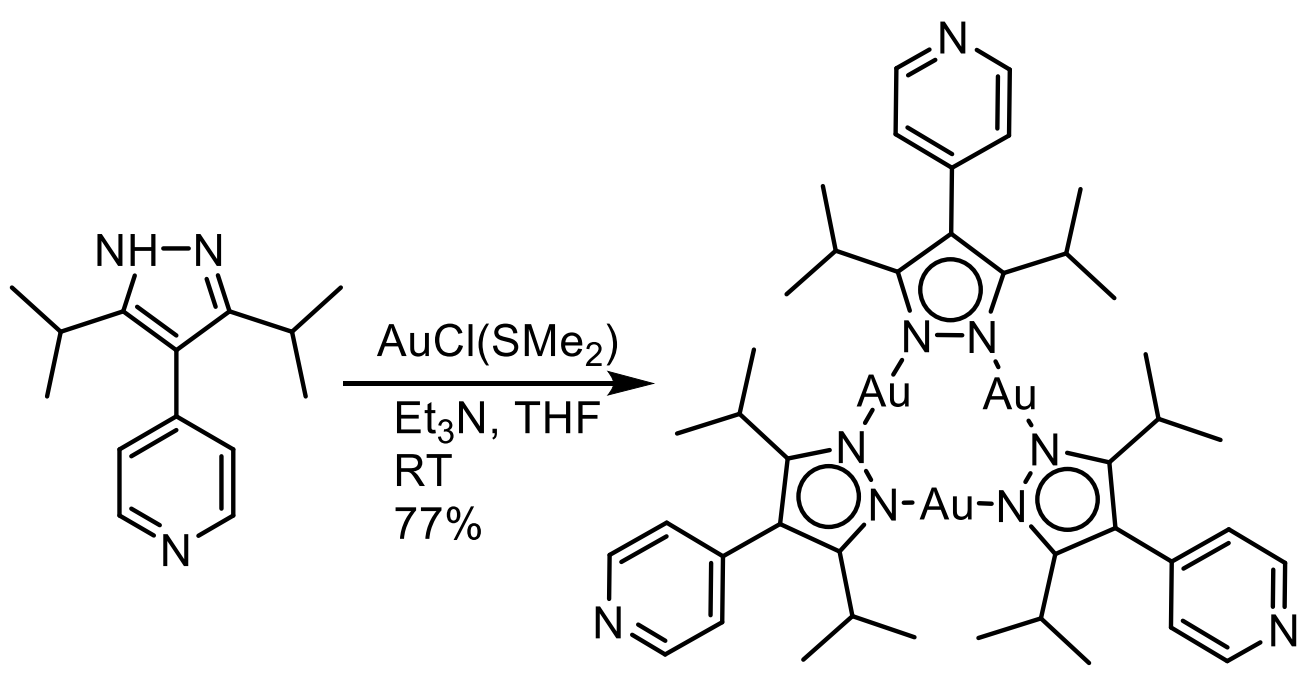

1

A solution of 4-(3,5-diisopropyl-1H-pyrazol-4-yl)pyridine (50 mg, 218 µmol, 1 eq.) was prepared in THF (10 mL) in a round bottom flask. NEt_3 (61 µL, 436 µmol, 2 eq.) was added and the mixture was stirred at RT for 5 min. $AuCl(SMe_2)$ (64 mg, 218 µmol, 1 eq.) was added and the mixture was stirred for 16 h at RT. The solvent was removed under reduced pressure and the crude was washed with MeOH (3 × 2 mL). The residue was dried under vacuum, dissolved in CH_2Cl_2, and filtered over celite. The solvent was removed under reduced pressure and the product was further dried under high vacuum to afford complex **1** as a white solid (yield: 71 mg, 77%). ^{1}H NMR (400 MHz, $CDCl_3$) δ 8.66 – 8.60 (m, 6H, H1), 7.22 – 7.16 (m, 6H, H2), 3.24 (p, *J* = 7.1 Hz, 6H, H3), 1.31 (d, *J* = 7.1 Hz, 36H, H4). ^{13}C{^{1}H} NMR (101 MHz, $CDCl_3$) δ 155.4, 149.7, 144.3, 126.8, 114.8, 28.1, 22.7, 22.6. HRMS (ESI/QTOF) *m/z*: [M + H]$^+$ Calcd for $C_{42}H_{55}Au_3N_9^+$ 1276.3572; Found 1276.3614.

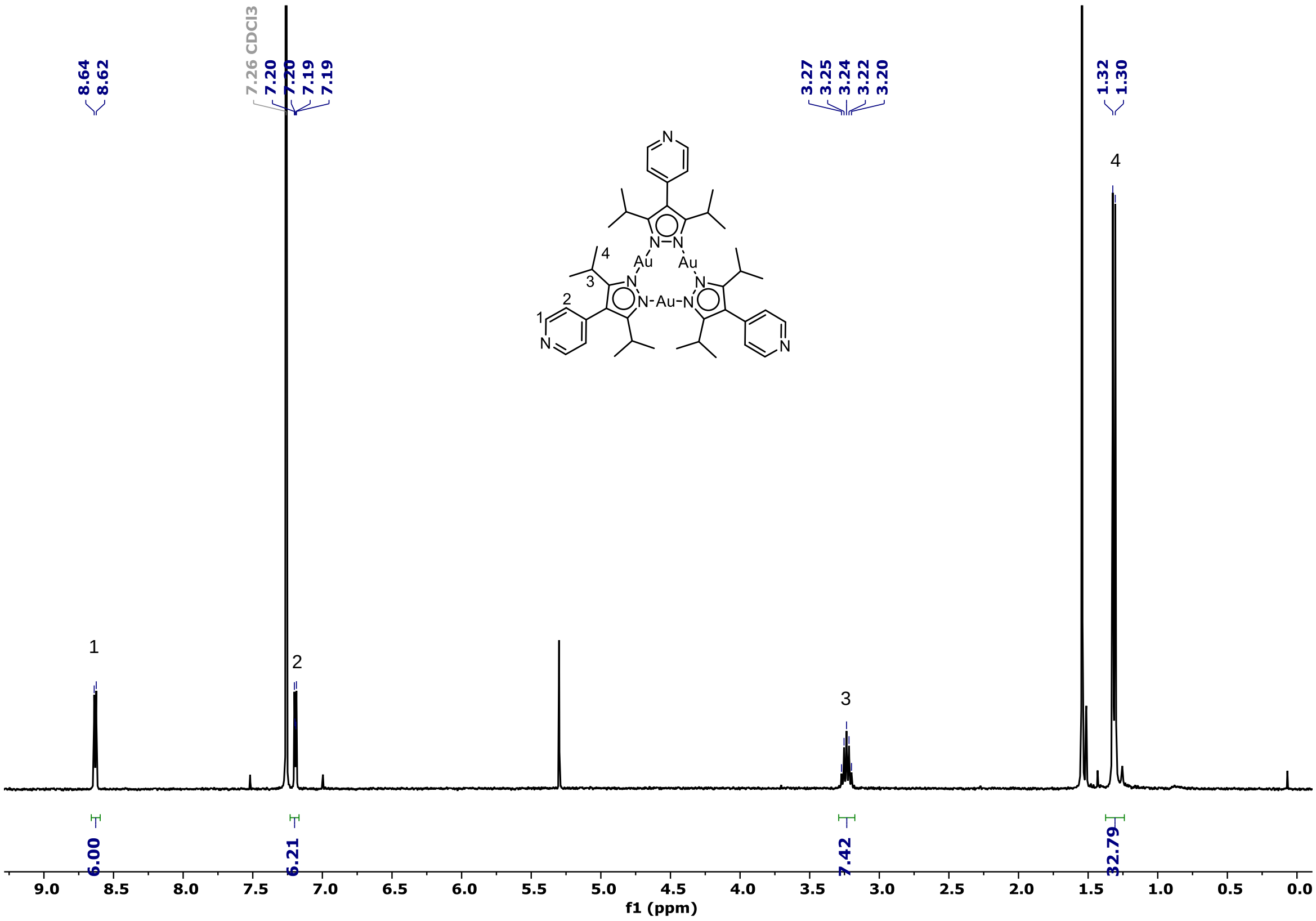

Figure S1. ^{1}H NMR spectrum ($CDCl_3$, 400 MHz) of complex **1**.

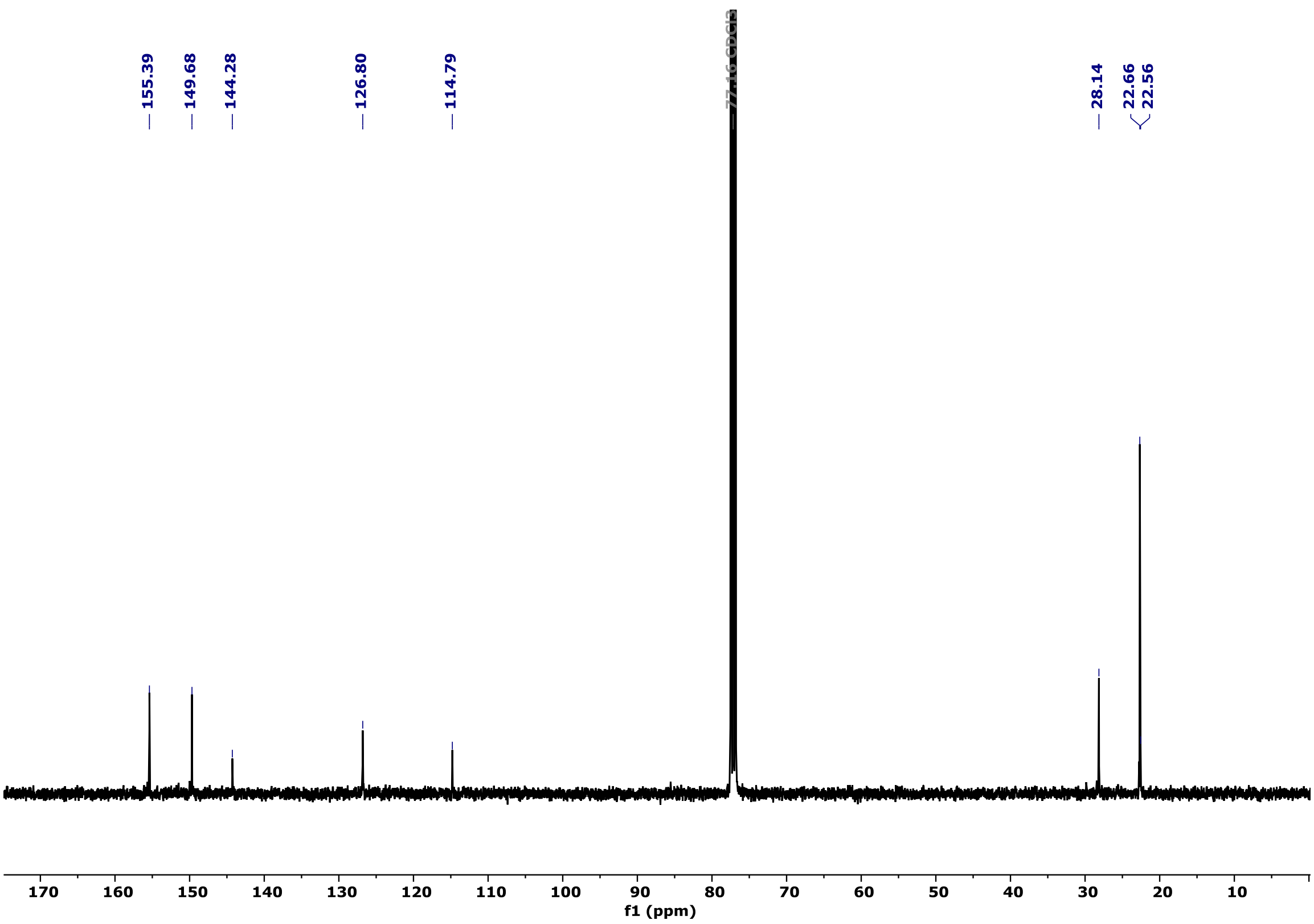

Figure S2. $^{13}C\{^1H\}$ NMR spectrum ($CDCl_3$, 101 MHz) of compound **1**.

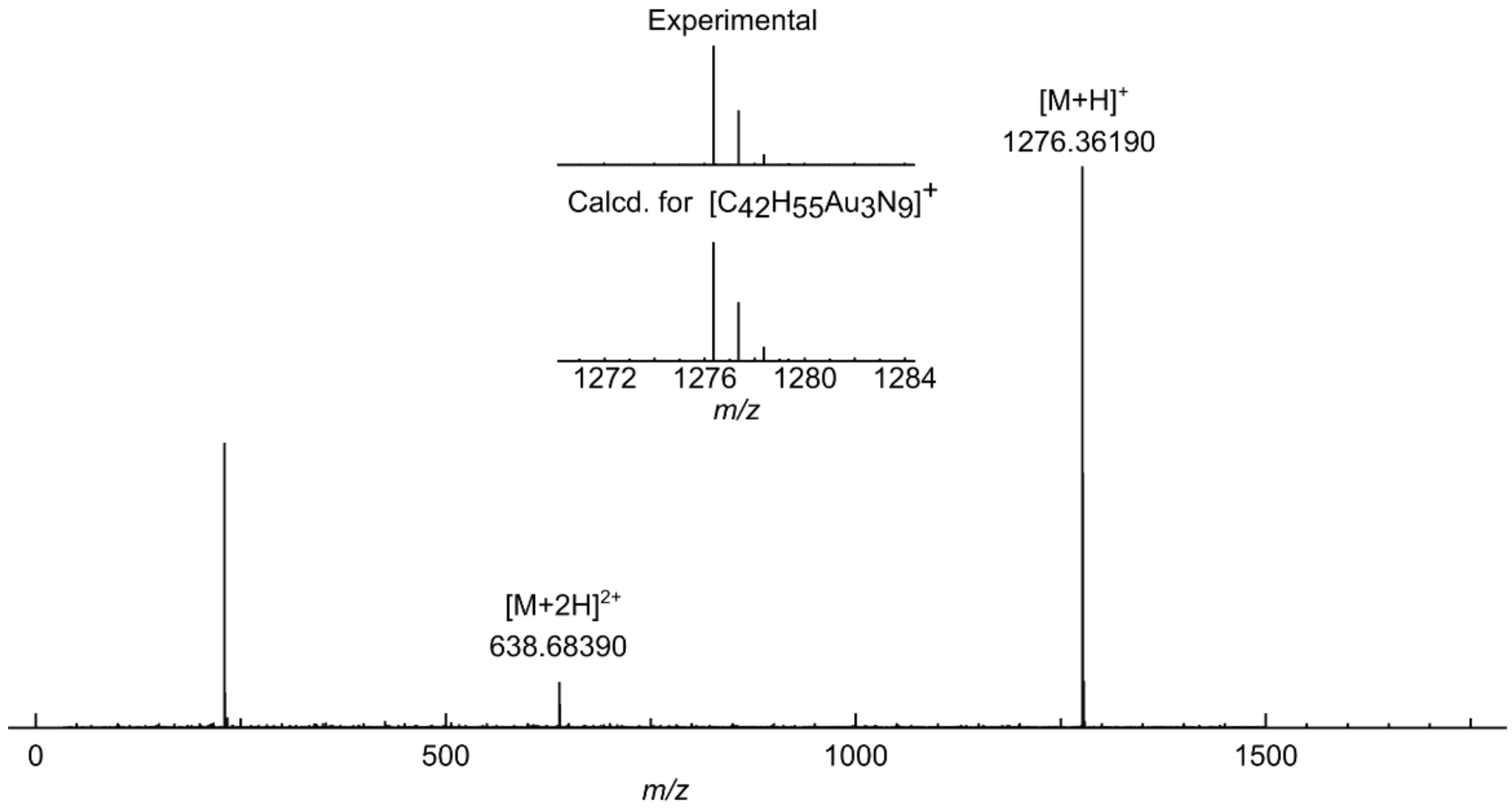

Figure S3. HRMS spectrum of complex **1**.

2.2 Synthesis of compound 2

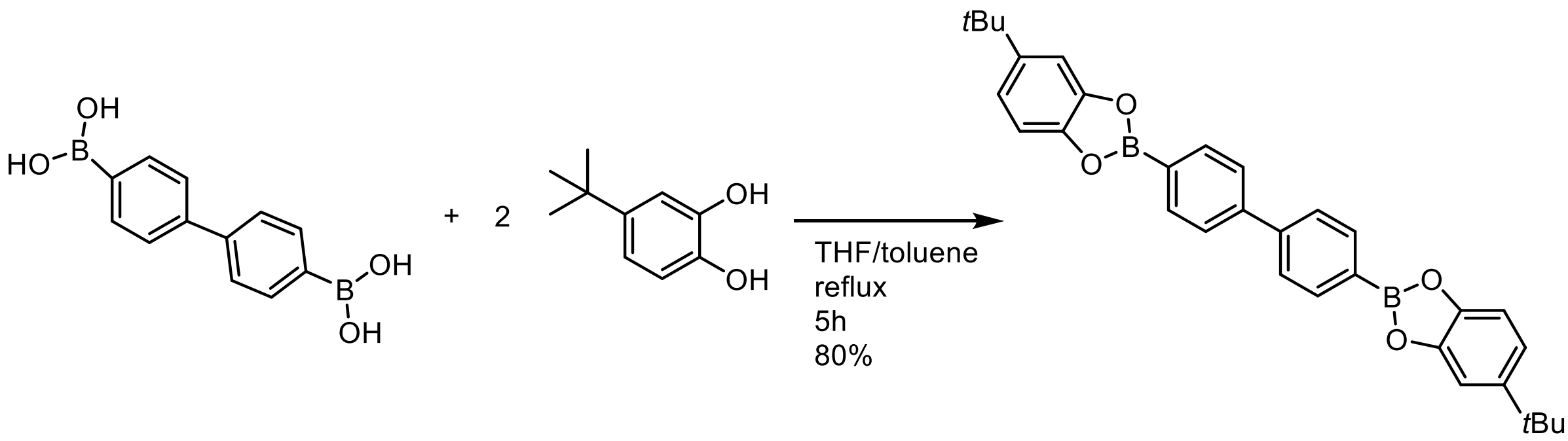

2

A solution of 4,4'-biphenyldiboronic acid (300 mg, 1.24 mmole, 1 eq.) and 4-tert-butylcatechol (412 mg, 2.48 mmole, 2 eq.) in a 2:1 toluene : THF mixture (80 mL) was heated under reflux for 5 h using a Dean Stark trap to remove water. The mixture was cooled to RT and the solvent was removed under reduced pressure. The crude compound was purified by recrystallization from toluene to afford the diboronate ester **2** as a white solid (yield: 500 mg, 80%). ^{1}H NMR (500 MHz, $CDCl_3$) δ 8.22 – 8.18 (m, 4H, H1), 7.83 – 7.79 (m, 4H, H2), 7.42 (d, *J* = 1.9 Hz, 2H, H3), 7.28 (d, *J* = 3.4 Hz, 2H, H4), 7.20 (dd, *J* = 8.4, 2.0 Hz, 2H, H5), 1.40 (s, 18H, H6). $^{13}C\{^{1}H\}$ NMR (126 MHz, $CDCl_3$) δ 148.6, 146.8, 146.4, 144.4, 135.66, 127.2, 119.7, 111.7, 110.1, 35.0, 31.9. HRMS (Sicrit plasma/LTQ-Orbitrap) *m/z*: $[M + H]^+$ Calcd for $C_{32}H_{33}B_2O_4^+$ 503.2559; Found 503.2553.

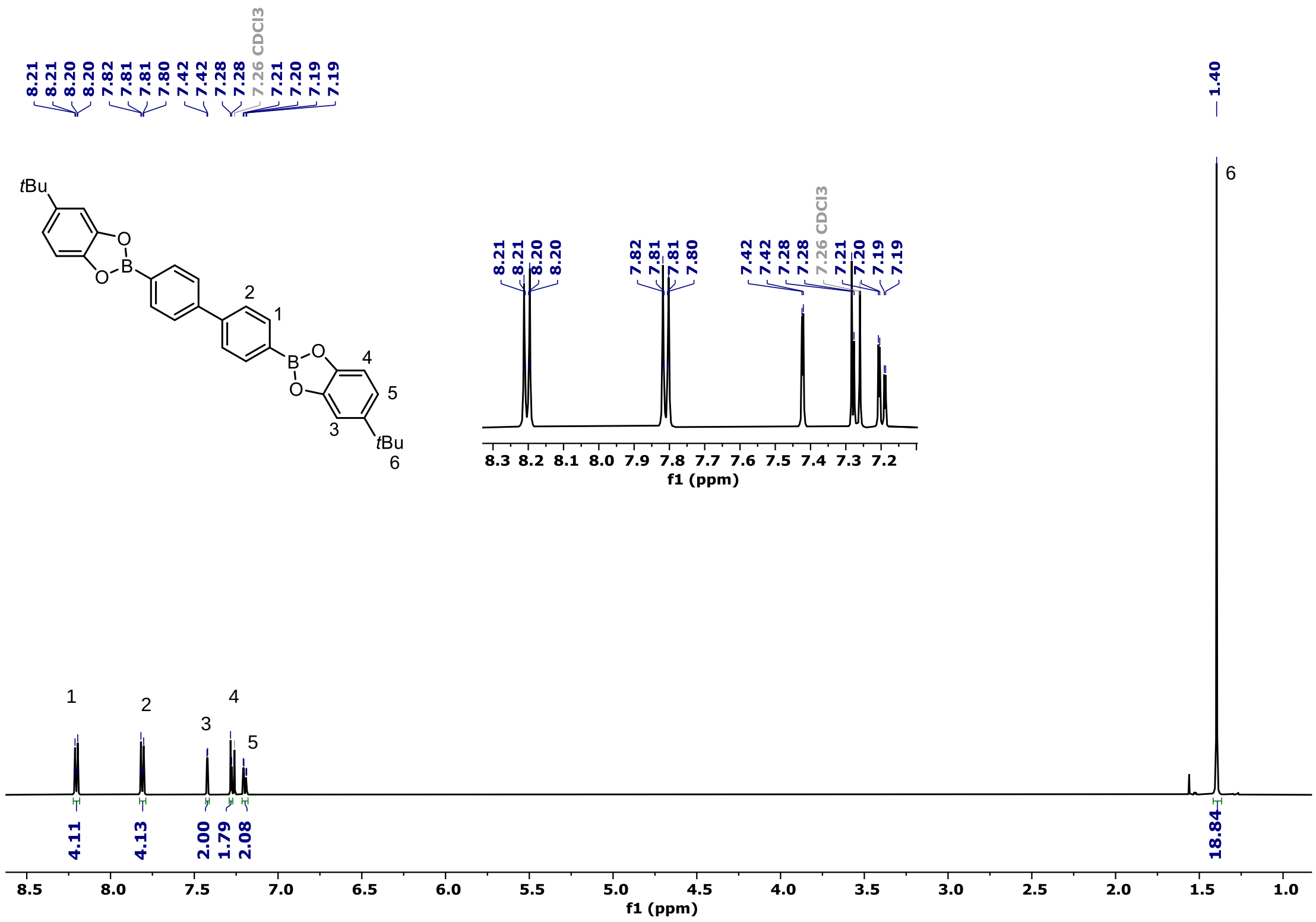

Figure S4. ^{1}H NMR spectrum ($CDCl_3$, 500 MHz) of compound **2**.

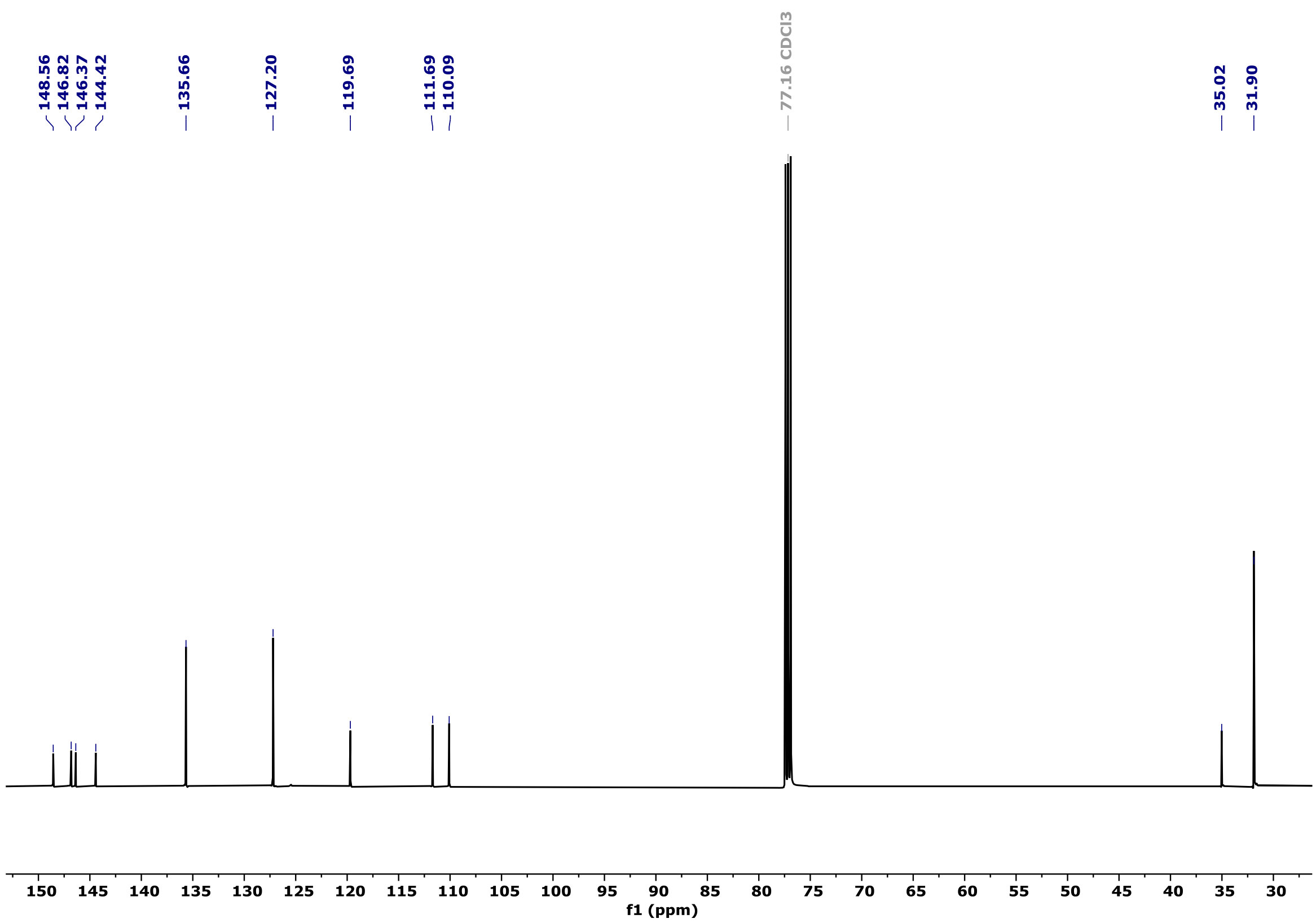

Figure S5. $^{13}C\{^{1}H\}$ NMR spectrum ($CDCl_3$, 126 MHz) of compound **2**.

2.3 Synthesis of crystalline B–N coordination polymer 3

A solution of complex **1** in anhydrous CH_2Cl_2 (150 µL, 4.18 mM, 1.0 eq.) was mixed under inert atmosphere with a solution of the diboronic ester **2** in anhydrous CH_2Cl_2 (150 µL, 6.28 mM, 1.5 eq.). Single crystals of **3** were grown through the diffusion of pentane vapors into this solution over the course of a week.

2.4 Synthesis of the crystalline 2D B–N network 6

A solution of complex **1** in anhydrous CH_2Cl_2 (150 µL, 4.18 mM, 1.0 eq.) was mixed under inert atmosphere with a solution of **4** in anhydrous CH_2Cl_2 (150 µL, 4.18 mM, 1.0 eq.). Single crystals of **6** were grown through the diffusion of pentane vapors into this solution over the course of a week.

2.5 Synthesis of the crystalline 2D B–N network 7

A solution of complex **1** in anhydrous CH_2Cl_2 (25 µL, 4.18 mM, 1.0 eq.) was mixed under inert atmosphere with *ortho*-dichlorobenzene (200 uL) and a solution of **5** in anhydrous CH_2Cl_2 (25 µL, 4.18 mM, 1.0 eq.). Single crystals of **7** were grown through the diffusion of pentane vapors into this solution over the course of a week.

2.7 Synthesis and activation of amorphous B–N networks

Complex **1** (21.12 mg, 16.55 µmol, 1 eq.) was dissolved in anhydrous CH_2Cl_2 (2 mL). Over this, a 2 mL CH_2Cl_2 solution containing 1 eq. boronic ester was added under inert atmosphere. The solvent was removed under reduced pressure and the yellow residue was dried under high vacuum for a few hours, followed by heating under vacuum between 80 and 120 °C for 16 h. The N_2 BET surface area was measured immediately after activation.

3. BET studies

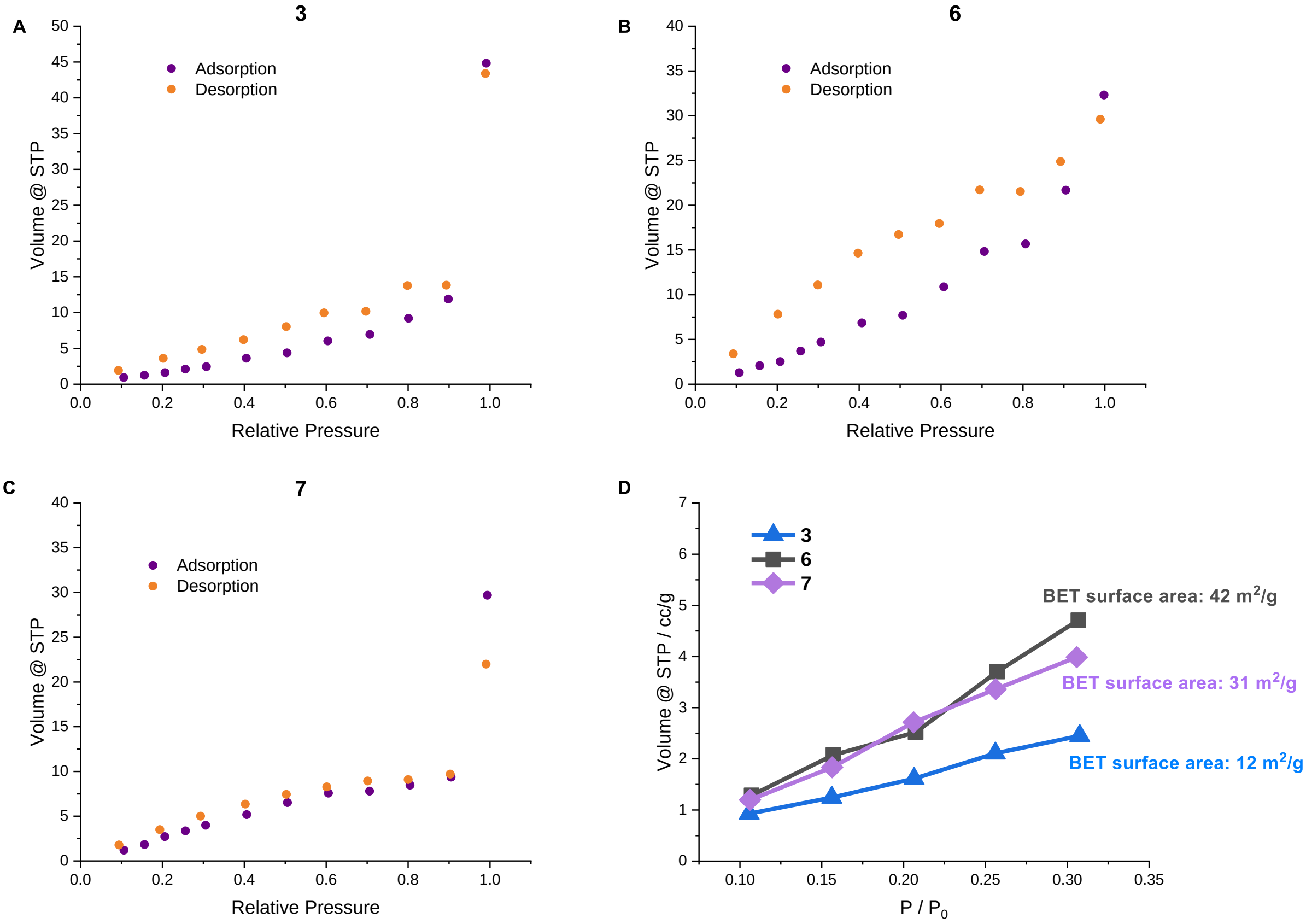

Figure S6. (A–**C**) N_2 adsorption and desorption isotherms of polymers **3**, **6**, and **7**, respectively, as determined by BET measurements performed at 77 K. (**D**) Comparison of the three polymers in the region ~0.10–0.30 P/P_0. These data were used for the determination of the multi-point BET surface-area values.

4. FT-IR of 6 and 7 before and after H_2O exposure

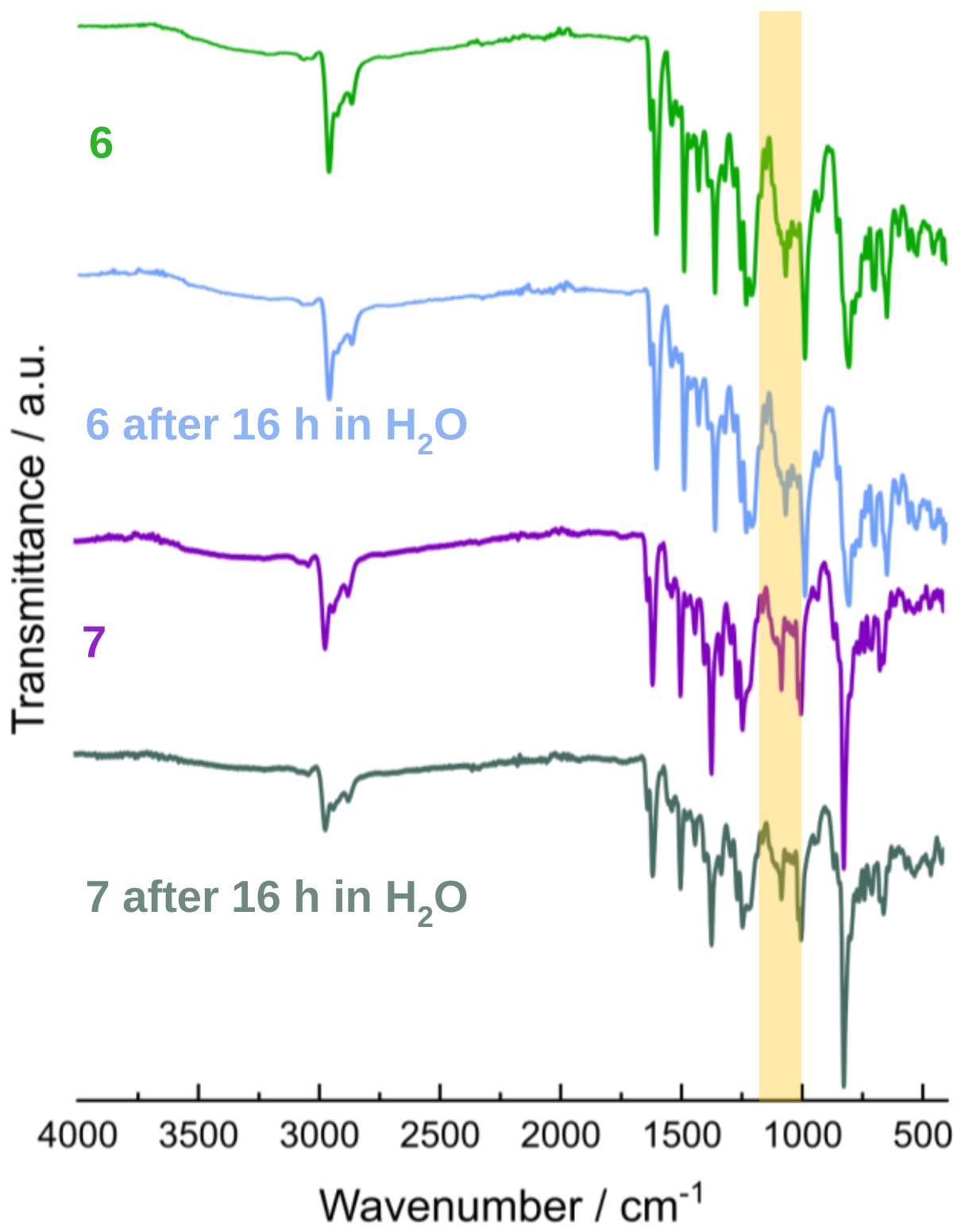

Figure S7. FT-IR spectra of **6** and **7** before and after exposure to H_2O for 16 h. The highlighted region indicates the typical range for B←N bond vibrations.[2]

5. TGA of amorphous powders of 6 and 7

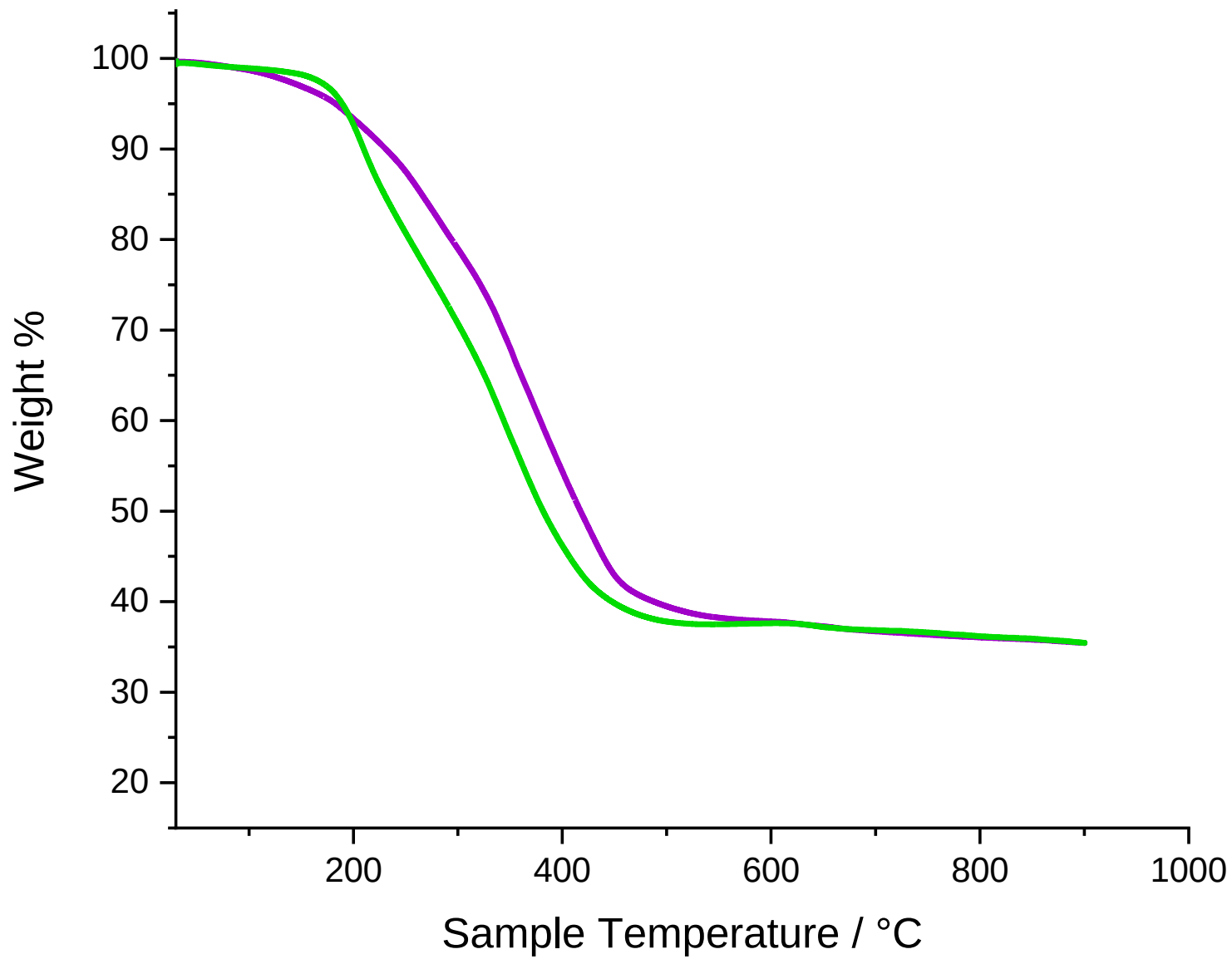

Figure S8. TGA of amorphous powders of **6** (green) and **7** (purple).

6. Single-crystal X-ray diffraction

6.1 Crystal data and experimental for 1

Experimental. Single colorless needle-shaped crystals of **1** were used as supplied. A suitable crystal with dimensions 0.17 × 0.02 × 0.01 mm was selected and mounted on a XtaLAB Synergy R, DW system, HyPix-Arc 150 diffractometer. The crystal was kept at a steady T = 229.99(10) K during data collection. The structure was solved with the ShelXT 2018/2[3]solution program using dual methods and by using Olex2 1.5[4] as the graphical interface. The model was refined with ShelXL 2019/3[5] using full matrix least squares minimization on $|F|^2$.

Crystal Data. $C_{48}H_{66}Au_3N_9O_2$, M_r = 1391.99, orthorhombic, *Fdd*2 (No. 43), a = 56.2726(6) Å, b = 40.4512(4) Å, c = 8.60408(7) Å, $\alpha = \beta = \gamma$ = 90°, V = 19585.4(3) Å^3, T = 229.99(10) K, Z = 16, Z' = 1, μ(Cu Kα) = 16.929, 28487 reflections measured, 8667 unique (R_{int} = 0.0561) which were used in all calculations. The final wR_2 was 0.1007 (all data) and R_1 was 0.0383 ($I \geq 2\sigma(I)$).

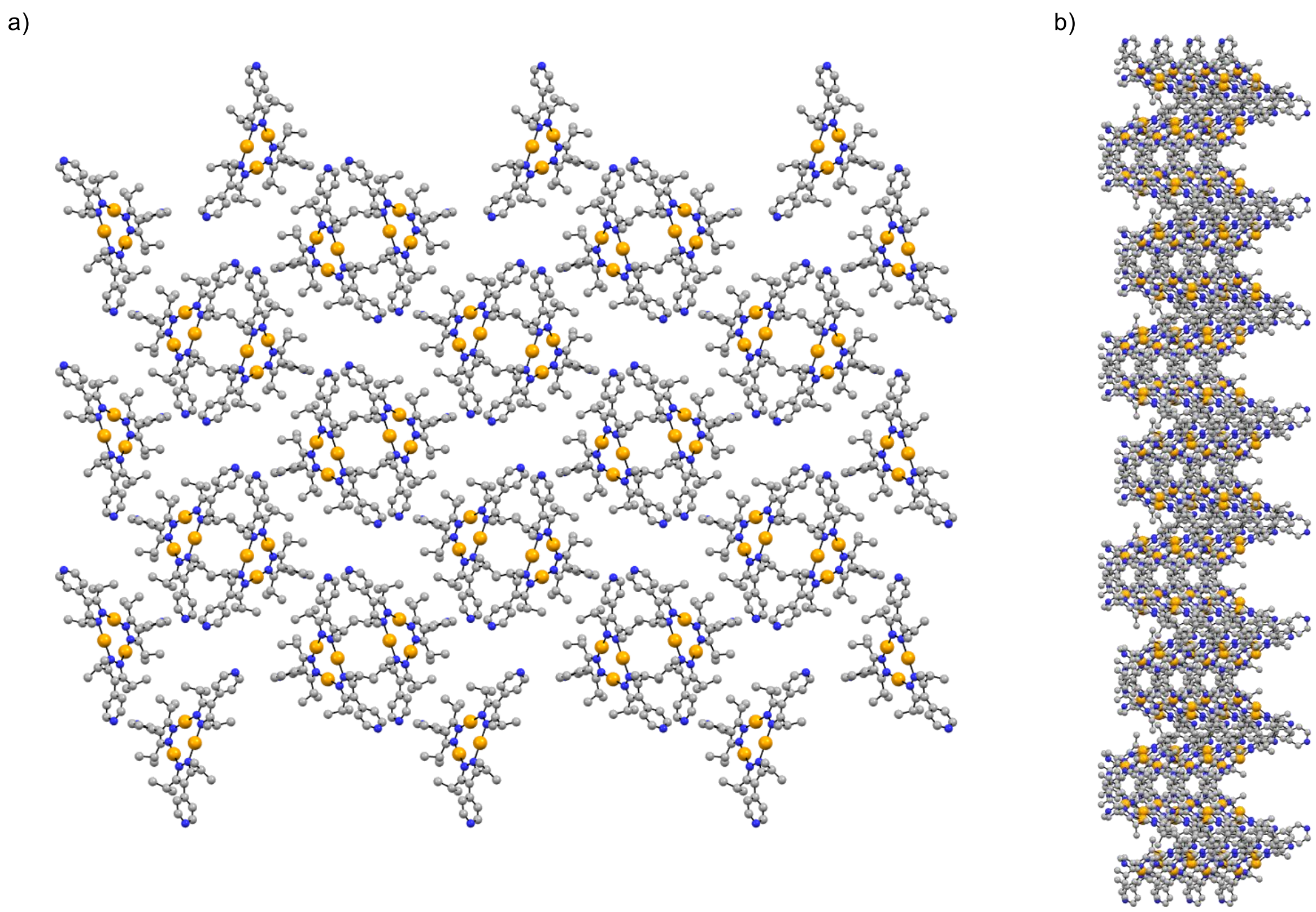

Figure S9. Packing of complex **1** in the crystal state, viewed along the c (**a**) and b axes (**b**). The hydrogen atoms were removed for clarity.

Table S1. Crystal data for **1**.

**Compound**	**1**
Formula	$C_{48}H_{66}Au_3N_9O_2$
$D_{calc.}$/ g cm^{-3}	1.888
μ/mm^{-1}	16.929
Formula Weight	1391.99
Color	colorless
Shape	needle-shaped
Size/mm	0.17 × 0.02 × 0.01
T/K	229.99(10)
Crystal System	orthorhombic
Flack Parameter	0.022(15)
Hooft Parameter	−0.014(7)
Space Group	*Fdd*2
a/Å	56.2726(6)
b/Å	40.4512(4)
c/Å	8.60408(7)
α/°	90
β/°	90
γ/°	90
V/Å^3	19585.4(3)
Z	16
Z'	1
Wavelength/Å	1.54184
Radiation type	Cu Kα
θ_{min}/°	3.827
θ_{max}/°	74.106
Index range h	$-67 \le h \le 67$
Index range k	$-37 \le k \le 48$
Index range l	$-10 \le l \le 9$
Measured Refl's.	28487
Indep't Refl's	8667
Refl's $I \ge 2\sigma(I)$	7889
R_{int}	0.0561
Parameters	498
Restraints	324
Largest Peak/e Å^{-3}	1.206
Deepest Hole/e Å^{-3}	−1.253
GooF	1.058
R_1 ($I \ge 2\sigma(I)$ / all)	0.0383 / 0.0428
wR_2 ($I \ge 2\sigma(I)$ / all)	0.0982 / 0.1007
CCDC number	2555662

6.2 Crystal data and experimental for 3

Experimental. Single colorless prism-shaped crystals of **3** were used as supplied. A suitable crystal with dimensions ? × 0.07 × 0.06 mm was selected and mounted on a XtaLAB Synergy R, DW system, HyPix-Arc 150 diffractometer. The crystal was kept at a steady T = 140.00(10) K during data collection. The structure was solved with the ShelXT 2018/2[3] solution program using dual methods and by using Olex2 1.5[4] as the graphical interface. The model was refined with ShelXL 2019/3[5] using full matrix least squares minimization on $|F|^2$.

Crystal Data. $C_{80}H_{98}Au_3B_2Cl_{12}N_9O_4$, M_r = 2287.59, monoclinic, *I*2/*a* (No. 15), *a* = 11.37557(19) Å, *b* = 28.5135(4) Å, *c* = 30.1756(8) Å, *β* = 91.696(2)°, *α* = *γ* = 90°, *V* = 9783.4(3) Å^3, *T* = 140.00(10) K, *Z* = 4, *Z'* = 0.5, *μ*(Cu K*α*) = 11.681, 46877 reflections measured, 9669 unique (R_{int} = 0.0365) which were used in all calculations. The final wR_2 was 0.2300 (all data) and R_1 was 0.0771 ($I \geq 2\sigma(I)$).

Table S2. Crystal data for **3**.

Compound	**3**
Formula	$C_{80}H_{98}Au_3B_2Cl_{12}N_9O_4$
$D_{calc.}$/ g cm^{-3}	1.553
μ/mm^{-1}	11.681
Formula Weight	2287.59
Color	colorless
Shape	prism-shaped
Size/mm	? × 0.07 × 0.06
T/K	140.00(10)
Crystal System	monoclinic
Space Group	*I*2/*a*
a/Å	11.37557(19)
b/Å	28.5135(4)
c/Å	30.1756(8)
α/°	90
β/°	91.696(2)
γ/°	90
V/Å^3	9783.4(3)
Z	4
Z'	0.5
Wavelength/Å	1.54184
Radiation type	Cu K*α*
θ_{min}/°	2.132
θ_{max}/°	75.217
Index range *h*	$-14 \leq h \leq 14$
Index range *k*	$-20 \leq k \leq 35$
Index range *l*	$-37 \leq l \leq 37$
Measured Refl's.	46877
Indep't Refl's	9669
Refl's $I \geq 2\sigma(I)$	7273
R_{int}	0.0365
Parameters	456
Restraints	769
Largest Peak/e Å^{-3}	3.153
Deepest Hole/e Å^{-3}	−1.537
GooF	1.069
R_1 ($I \geq 2\sigma(I)$ / all)	0.0771 / 0.0905
wR_2 ($I \geq 2\sigma(I)$ / all)	0.2201 / 0.2300
CCDC number	2555663

6.3 Crystal data and experimental for fluorobenzene⊂3

Experimental. Single crystals of **3**, which were obtained as previously described, were soaked in pure fluorobenzene for 24 h to give single, colorless irregular-shaped crystals of **fluorobenzene⊂3** . A suitable crystal with dimensions 0.09 × 0.07 × 0.03 mm was selected and mounted on a XtaLAB Synergy R, DW system, HyPix-Arc 150 diffractometer. The crystal was kept at a steady *T* = 140.00(10)

K during data collection. The structure was solved with the ShelXT 2018/2[3] solution program using dual methods and by using Olex2 1.5[4] as the graphical interface. The model was refined with ShelXL 2019/3[5] using full matrix least squares minimization on $|F|^2$.

Crystal Data. $C_{110}H_{116}Au_3B_2F_6N_9O_4$, M_r = 2354.63, monoclinic, $I2/a$ (No. 15), a = 11.63009(10) Å, b = 27.49661(18) Å, c = 33.6992(4) Å, β = 98.7413(10)°, $\alpha = \gamma$ = 90°, V = 10651.42(18) Å^3, T = 140.00(10) K, Z = 4, Z' = 0.5, μ(Cu Kα) = 8.131, 93948 reflections measured, 10764 unique (R_{int} = 0.0264) which were used in all calculations. The final wR_2 was 0.1858 (all data) and R_1 was 0.0594 ($I \geq 2\sigma(I)$).

Table S3. Crystal data for **fluorobenzene⊂3**.

Compound	**fluorobenzene⊂3**
Formula	$C_{110}H_{116}Au_3B_2F_6N_9O_4$
$D_{calc.}$/ g cm^{-3}	1.468
m/mm^{-1}	8.131
Formula Weight	2354.63
Color	colorless
Shape	irregular-shaped
Size/mm	0.09×0.07×0.03
T/K	140.00(10)
Crystal System	monoclinic
Space Group	*I*2/*a*
a/Å	11.63009(10)
b/Å	27.49661(18)
c/Å	33.6992(4)
a/°	90
b/°	98.7413(10)
g/°	90
V/Å^3	10651.42(18)
Z	4
Z'	0.5
Wavelength/Å	1.54184
Radiation type	Cu K_a
Q_{min}/°	2.084
Q_{max}/°	74.717
Index range h	-14 ≤ h ≤ 14
Index range k	-23 ≤ k ≤ 34
Index range l	-41 ≤ l ≤ 42
Measured Refl's.	93948
Indep't Refl's	10764
Refl's I≥2*s*(I)	8964
R_{int}	0.0264
Parameters	598
Restraints	1164
Largest Peak/eÅ^3	1.041
Deepest Hole/eÅ^3	-1.959
GooF	1.043
R_1 (I≥2*s*(I) / all)	0.0594 / 0.0674
wR_2 (I≥2*s*(I) / all)	0.1781 / 0.1858
CCDC number	2555664

6.4 Crystal data and experimental for 6

Experimental. Single colorless irregular-shaped crystals of **6** were used as supplied. A suitable crystal with dimensions 0.06 × 0.06 × 0.05 mm was selected and mounted on a XtaLAB Synergy R, DW system, HyPix-Arc 150 diffractometer. The crystal was kept at a steady T = 140.00(10) K during data collection. The structure was solved with the ShelXT 2018/2[3] solution program using dual methods and by using Olex2 1.5[4] as the graphical interface. The model was refined with ShelXL 2019/3[5] using full matrix least squares minimization on $|F|^2$.

Crystal Data. $C_{117}H_{156}Au_3B_3Cl_6N_9O_{10.5}$, M_r = 2692.53, trigonal, $R3$ (No. 146), a = 26.0811(3) Å, b = 26.0811(3) Å, c = 16.38031(19) Å, α = 90°, β = 90°, γ = 120°, V = 9649.5(2) Å^3, T = 140.00(10) K, Z = 3, Z' = 1/3, μ(Cu Kα) = 7.885, 45317 reflections measured, 8237 unique (R_{int} = 0.0289) which were used in all calculations. The final wR_2 was 0.1083 (all data) and R_1 was 0.0397 ($I \geq 2\sigma(I)$).

Table S4. Crystal data for **6**.

Compound	**6**
Formula	$C_{117}H_{156}Au_3B_3Cl_6N_9O_{10.5}$
$D_{calc.}$/ g cm^{-3}	1.390
μ/mm^{-1}	7.885
Formula Weight	2692.53
Color	colorless
Shape	irregular-shaped
Size/mm	0.06 × 0.06 × 0.05
T/K	140.00(10)
Crystal System	trigonal
Flack Parameter	0.001(10)
Hooft Parameter	0.064(2)
Space Group	$R3$
a/Å	26.0811(3)
b/Å	26.0811(3)
c/Å	16.38031(19)
α/°	90
β/°	90
γ/°	120
V/Å^3	9649.5(2)
Z	3
Z'	1/3
Wavelength/Å	1.54184
Radiation type	Cu Kα
θ_{min}/°	3.333
θ_{max}/°	75.562
Index range h	$-31 \leq h \leq 32$
Index range k	$-32 \leq k \leq 32$
Index range l	$-20 \leq l \leq 19$
Measured Refl's.	45317
Indep't Refl's	8237
Refl's $I \geq 2\sigma(I)$	7521
R_{int}	0.0289
Parameters	373
Restraints	81
Largest Peak/e Å^{-3}	1.255
Deepest Hole/e Å^{-3}	−0.955
GooF	1.054
R_1 ($I \geq 2\sigma(I)$ / all)	0.0397 / 0.0441
wR_2 ($I \geq 2\sigma(I)$ / all)	0.1056 / 0.1083
CCDC number	2555665

6.5 Crystal data and experimental for fluorobenzene⊂6

Experimental. Single crystals of **6**, which were obtained as previously described, were soaked in pure fluorobenzene for 24 h to give single clear light yellow prism-shaped crystals of **fluorobenzene⊂6.** A suitable crystal with dimensions 0.16 × 0.09 × 0.08 mm was selected and mounted on a XtaLAB Synergy R, DW system, HyPix-Arc 150 diffractometer. The crystal was kept at a steady T = 100.00(10) K during data collection. The structure was solved with the ShelXT 2018/2[3] solution program using dual methods and by using Olex2 1.5[4] as the graphical interface. The model was refined with ShelXL 2019/3[5] using full matrix least squares minimization on $|F|^2$.

Crystal Data. $C_{129}H_{132.5}Au_3B_3F_{5.5}N_9O_6$, M_r = 2632.76, hexagonal, $P6_1$ (No. 169), a = 26.6519(5) Å, b = 26.6519(5) Å, c = 30.1466(6) Å, α = 90°, β = 90°, γ = 120°, V = 18544.9(8) Å^3, T = 100.00(10) K, Z = 6, Z' = 1, μ(Cu Kα) = 7.073, 69559 reflections measured, 12785 unique (R_{int} = 0.0535) which were used in all calculations. The final wR_2 was 0.2104 (all data) and R_1 was 0.0750 ($I \geq 2\sigma(I)$).

Table S5. Crystal data for **fluorobenzene⊂6**.

Compound	**fluorobenzene⊂6**
Formula	$C_{129}H_{132.5}Au_3B_3F_{5.5}N_9O_6$
$D_{calc.}$/ g cm^{-3}	1.414
μ/mm^{-1}	7.073
Formula Weight	2632.76
Color	clear light yellow
Shape	prism-shaped
Size/mm	0.16 × 0.09 × 0.08
T/K	100.00(10)
Crystal System	hexagonal
Flack Parameter	0.047(18)
Hooft Parameter	0.057(3)
Space Group	$P6_1$
a/Å	26.6519(5)
b/Å	26.6519(5)
c/Å	30.1466(6)
α/°	90
β/°	90
γ/°	120
V/Å^3	18544.9(8)
Z	6
Z'	1
Wavelength/Å	1.54184
Radiation type	Cu Kα
θ_{min}/°	1.914
θ_{max}/°	51.523
Index range h	$-26 \le h \le 26$
Index range k	$-24 \le k \le 26$
Index range l	$-27 \le l \le 30$
Measured Refl's.	69559
Indep't Refl's	12785
Refl's $I \ge 2\sigma(I)$	10269
R_{int}	0.0535
Parameters	1166
Restraints	1943
Largest Peak/e Å^{-3}	1.929
Deepest Hole/e Å^{-3}	−0.518
GooF	1.102
R_1 ($I \ge 2\sigma(I)$ / all)	0.0750 / 0.0907
wR_2 ($I \ge 2\sigma(I)$ / all)	0.2000 / 0.2104
CCDC number	2555667

6.6 Crystal data and experimental for chlorobenzene⊂6

Experimental Single crystals of **6**, which were obtained as previously described, were soaked in pure chlorobenzene for 24 h to give single clear light-yellow prism-shaped crystals of **chlorobenzene⊂6**. A suitable crystal with dimensions 0.18 × 0.04 × 0.01 mm was selected and mounted on a XtaLAB Synergy R, DW system, HyPix-Arc 150 diffractometer. The crystal was kept at a steady T = 139.99(10) K during data collection. The structure was solved with the ShelXT 2018/2[3] solution program using dual methods and by using Olex2 1.5[4] as the graphical interface. The model was refined with ShelXL 2019/3[5] using full matrix least squares minimization on $|F|^2$.

Crystal Data. $C_{132}H_{135}Au_3B_3Cl_6N_9O_6$, M_r = 2779.51, hexagonal, $P6_5$ (No. 170), a = 26.6243(3) Å, b = 26.6243(3) Å, c = 30.3971(6) Å, α = 90°, β = 90°, γ = 120°, V = 18660.3(6) Å3, T = 139.99(10) K, Z = 6, Z' = 1, μ(Cu K_α) = 8.154, 103306 reflections measured, 17687 unique (R_{int} = 0.0561) which were used in all calculations. The final wR_2 was 0.1729 (all data) and R_1 was 0.0627 (I≥2σ(I)).

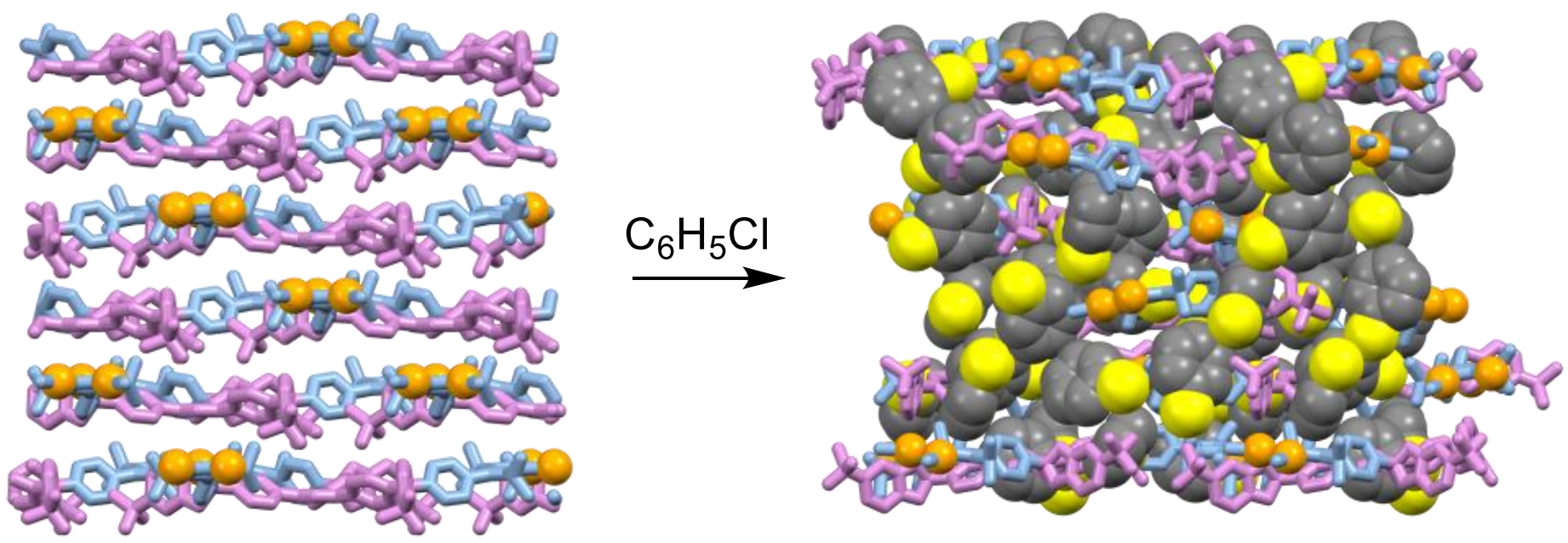

6 **chlorobenzene⊂6**

Figure S10. Graphic representations of the structure of network **6** before and after exposure to chlorobenzene. Hydrogen atoms and solvent molecules in **6** are not shown.

Table S6. Crystal data for **chlorobenzene⊂6**.

**Compound**	**chlorobenzene⊂6**
Formula	$C_{132}H_{135}Au_3B_3Cl_6N_9O_6$
$D_{calc.}$/ g cm^{-3}	1.484
μ/mm^{-1}	8.154
Formula Weight	2779.51
Colour	colourless
Shape	plate-shaped
Size/mm	0.18×0.04×0.01
T/K	139.99(10)
Crystal System	hexagonal
Flack Parameter	-0.009(5)
Hooft Parameter	-0.006(3)
Space Group	$P6_5$
a/Å	26.6243(3)
b/Å	26.6243(3)
c/Å	30.3971(6)
α/°	90
β/°	90
γ/°	120
V/Å^3	18660.3(6)
Z	6
Z'	1
Wavelength/Å	1.54184
Radiation type	Cu K_α
θ_{min}/°	1.916
θ_{max}/°	58.927
Index range h	$-29 \leq h \leq 28$
Index range k	$-29 \leq k \leq 22$
Index range l	$-33 \leq l \leq 33$
Measured Refl's.	103306
Indep't Refl's	17687
Refl's I≥2σ(I)	13440
R_{int}	0.0561
Parameters	1615
Restraints	3088
Largest Peak/eÅ^3	1.478
Deepest Hole/eÅ^3	-0.949
GooF	1.017
R_1 (I≥2σ(I) / all)	0.0627 / 0.0880
wR_2 (I≥2σ(I) / all)	0.1581 / 0.1729
CCDC number	2575900

6.7 Crystal structure of bromobenzene⊂6

Experimental Single crystals of **6**, which were obtained as previously described, were soaked in pure bromobenzene for 24 h to give single clear light yellow prism-shaped crystals of **bromobenzene⊂6**. A

suitable crystal with dimensions 0.16 × 0.09 × 0.08 mm^3 was selected and mounted on a XtaLAB Synergy R, DW system, HyPix-Arc 150 diffractometer. The crystal was kept at a steady T = 100.00(10) K during data collection. The structure was solved with the **ShelXT** 2018/2[3] solution program using dual methods and by using **Olex2** 1.5[4] as the graphical interface. The model was refined with **ShelXL** 2018/3[5] using full matrix least squares minimization on $\boldsymbol{F^2}$.

Crystal data. $C_{132}H_{135}Au_3B_3Br_6N_9O_6$, M_r = 3046.27, hexagonal, $P6_1$ (No. 169), a = 26.6322(4) Å, b = 26.6322(4) Å, c = 30.6146(6) Å, α = 90˚, β = 90˚, γ = 120˚, V = 18804.9(6) Å^3, T = 100.00(10) K, Z = 6, Z' = 1, μ(Cu K_α) = 9.156, 74144 reflections measured, 7582 unique (R_{int} = 0.0828) which were used in all calculations. The final wR_2 was 0.2020 (all data) and R_1 was 0.0723 (I≥2 σ(I)).

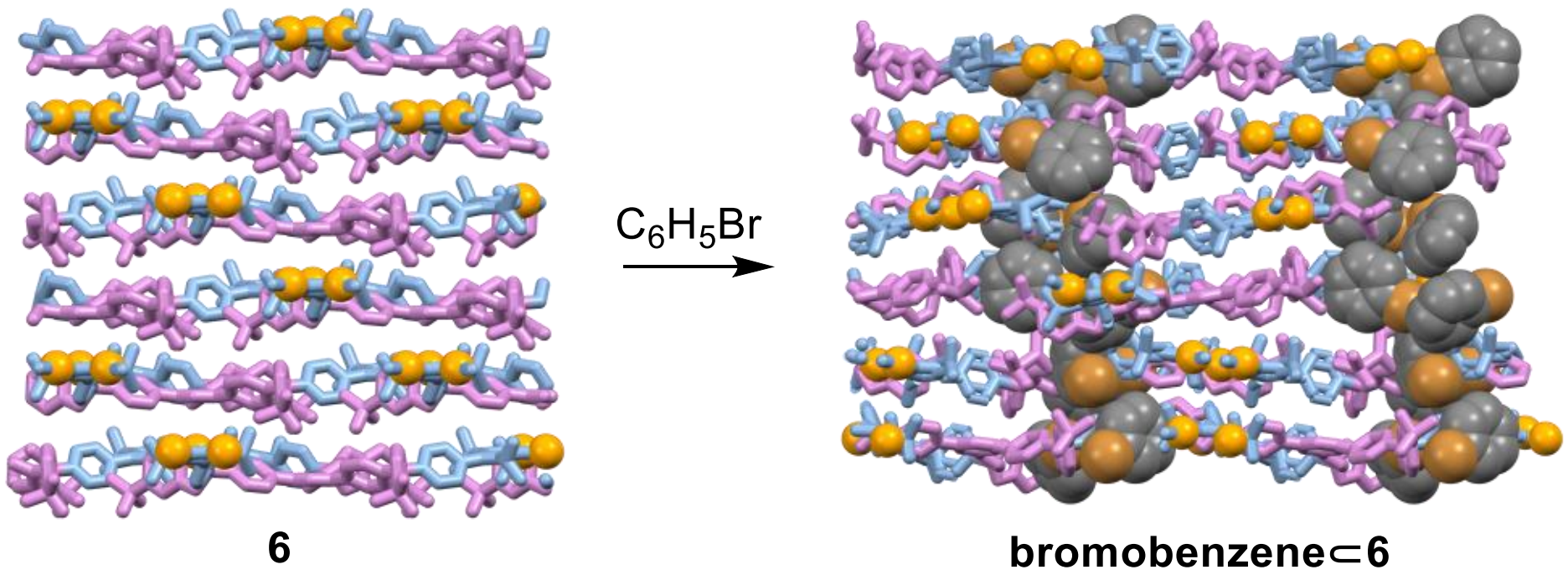

Figure S11. Graphic representations of the structure of network **6** before and after exposure to bromobenzene. Hydrogen atoms and solvent molecules in **6** are not shown.

Table S7. Crystal data for bromobenzene⊂6.

**Compound**	**bromobenzene⊂6.**
Formula	$C_{132}H_{135}Au_3B_3Br_6N_9O_6$
$D_{calc.}$/ g cm^{-3}	1.614
μ/mm^{-1}	9.156
Formula Weight	3046.27
Colour	colourless
Shape	needle-shaped
Size/mm^3	0.16×0.09×0.08
T/K	100.00(10)
Crystal System	hexagonal
Flack Parameter	0.12(3)
Space Group	*P*6$_1$
a/Å	26.6322(4)
b/Å	26.6322(4)
c/Å	30.6146(6)
α/°	90
β/°	90
γ/°	120
V/Å^3	18804.9(6)
Z	6
Z'	1
Wavelength/Å	1.54184
Radiation type	Cu K_α
θ_{min}/°	2.398
θ_{max}/°	39.968
Measured Refl's.	74144
Indep't Refl's	7582
Refl's I≥2 σ(I)	6345
R_{int}	0.0828
Parameters	1041
Restraints	2049
Largest Peak	1.322
Deepest Hole	-0.658
GooF	1.062
wR_2 (all data)	0.2020
wR_2	0.1909
R_1 (all data)	0.0863
R_1	0.0723
CCDC number	2575901

6.8 Crystal data and experimental for 7

Experimental. Single colourless prism-shaped crystals of **7** were used as supplied. A suitable crystal with dimensions 0.17 × 0.07 × 0.04 mm was selected and mounted on a XtaLAB Synergy R, DW system, HyPix-Arc 150 diffractometer. The crystal was kept at a steady *T* = 140.00(10) K during data collection. The structure was solved with the ShelXT 2018/2[3] solution program using dual methods and by using Olex2 1.5[4] as the graphical interface. The model was refined with ShelXL 2019/3[5] using full matrix least squares minimisation on $|F|^2$.

Crystal Data. $C_{147}H_{137}Au_3B_3Cl_{11}N_9O_6$, M_r = 3138.93, monoclinic, *I*2/*a* (No. 15), *a* = 23.8106(5) Å, *b* = 30.8765(4) Å, *c* = 43.1091(7) Å, β = 103.1833(17)°, $\alpha = \gamma$ = 90°, *V* = 30858.0(9) Å^3, *T* = 140.00(10)

K, Z = 8, Z' = 1, μ(Cu Kα) = 7.419, 74928 reflections measured, 16601 unique (R_{int} = 0.0462) which were used in all calculations. The final wR_2 was 0.2713 (all data) and R_1 was 0.0791 ($I \geq 2\sigma(I)$).

Table S8. Crystal data for **7**.

Compound	**7**
Formula	$C_{147}H_{137}Au_3B_3Cl_{11}N_9O_6$
$D_{calc.}$/ g cm^{-3}	1.351
μ/mm^{-1}	7.419
Formula Weight	3138.93
Colour	colourless
Shape	prism-shaped
Size/mm	0.17 × 0.07 × 0.04
T/K	140.00(10)
Crystal System	monoclinic
Space Group	$I2/a$
a/Å	23.8106(5)
b/Å	30.8765(4)
c/Å	43.1091(7)
α/°	90
β/°	103.1833(17)
γ/°	90
V/Å^3	30858.0(9)
Z	8
Z'	1
Wavelength/Å	1.54184
Radiation type	Cu Kα
θ_{min}/°	1.776
θ_{max}/°	51.505
Index range h	$-23 \leq h \leq 18$
Index range k	$-31 \leq k \leq 30$
Index range l	$-43 \leq l \leq 43$
Measured Refl's.	74928
Indep't Refl's	16601
Refl's $I \geq 2\sigma(I)$	10470
R_{int}	0.0462
Parameters	1126
Restraints	1882
Largest Peak/e Å^{-3}	2.780
Deepest Hole/e Å^{-3}	−0.795
GooF	1.055
R_1 ($I \geq 2\sigma(I)$ / all)	0.0791 / 0.1077
wR_2 ($I \geq 2\sigma(I)$ / all)	0.2529 / 0.2713
CCDC number	2555666

7. Powder X-Ray Diffraction

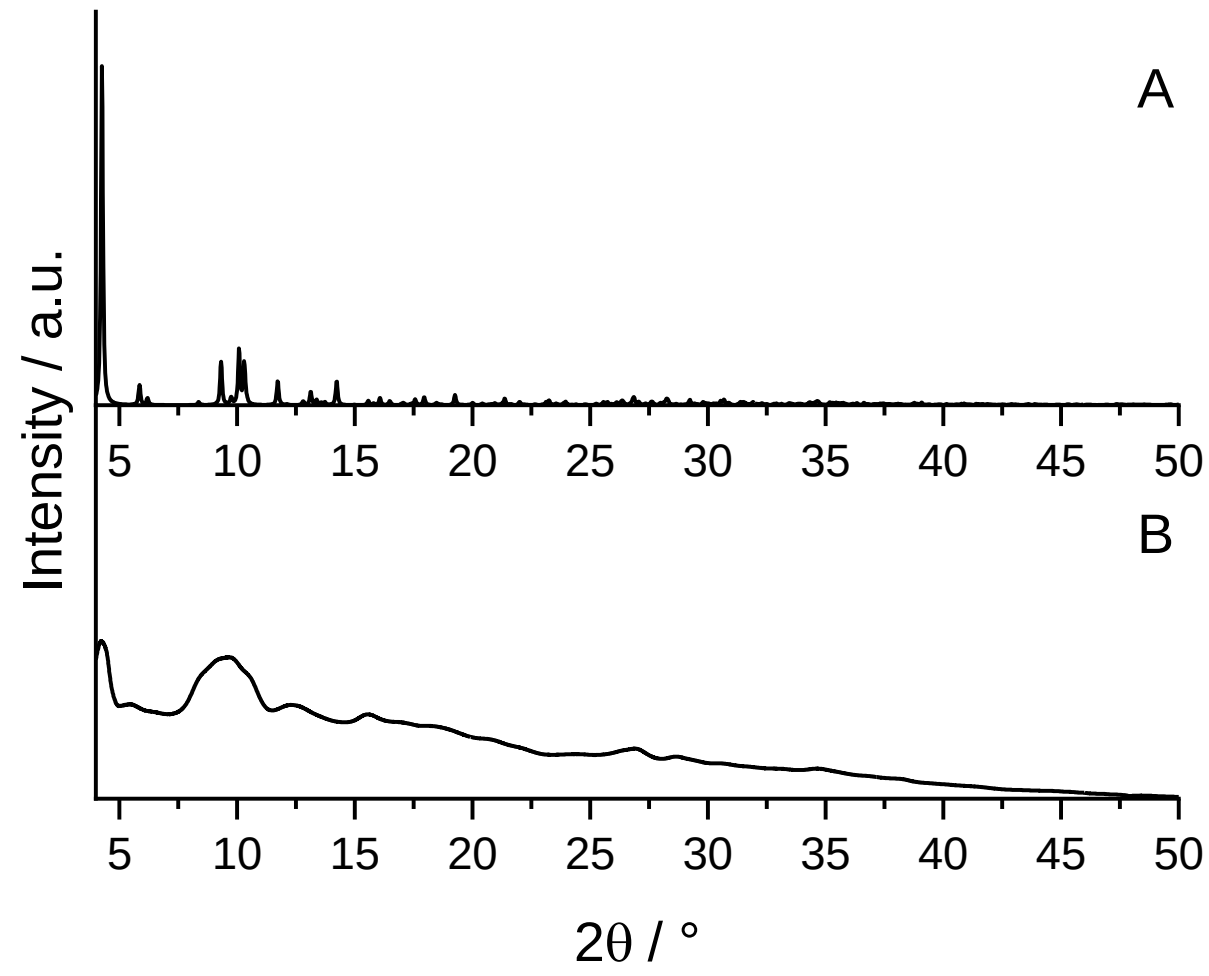

Figure S12. PXRD diffractograms of polymer **3**. **A**: Simulated PXRD pattern calculated from the single-crystal X-ray structure. **B**: Experimental PXRD pattern of single crystals of **3**. The measurement was performed immediately after removal of the crystals from the mother liquor. The data indicate rapid loss of crystallinity.

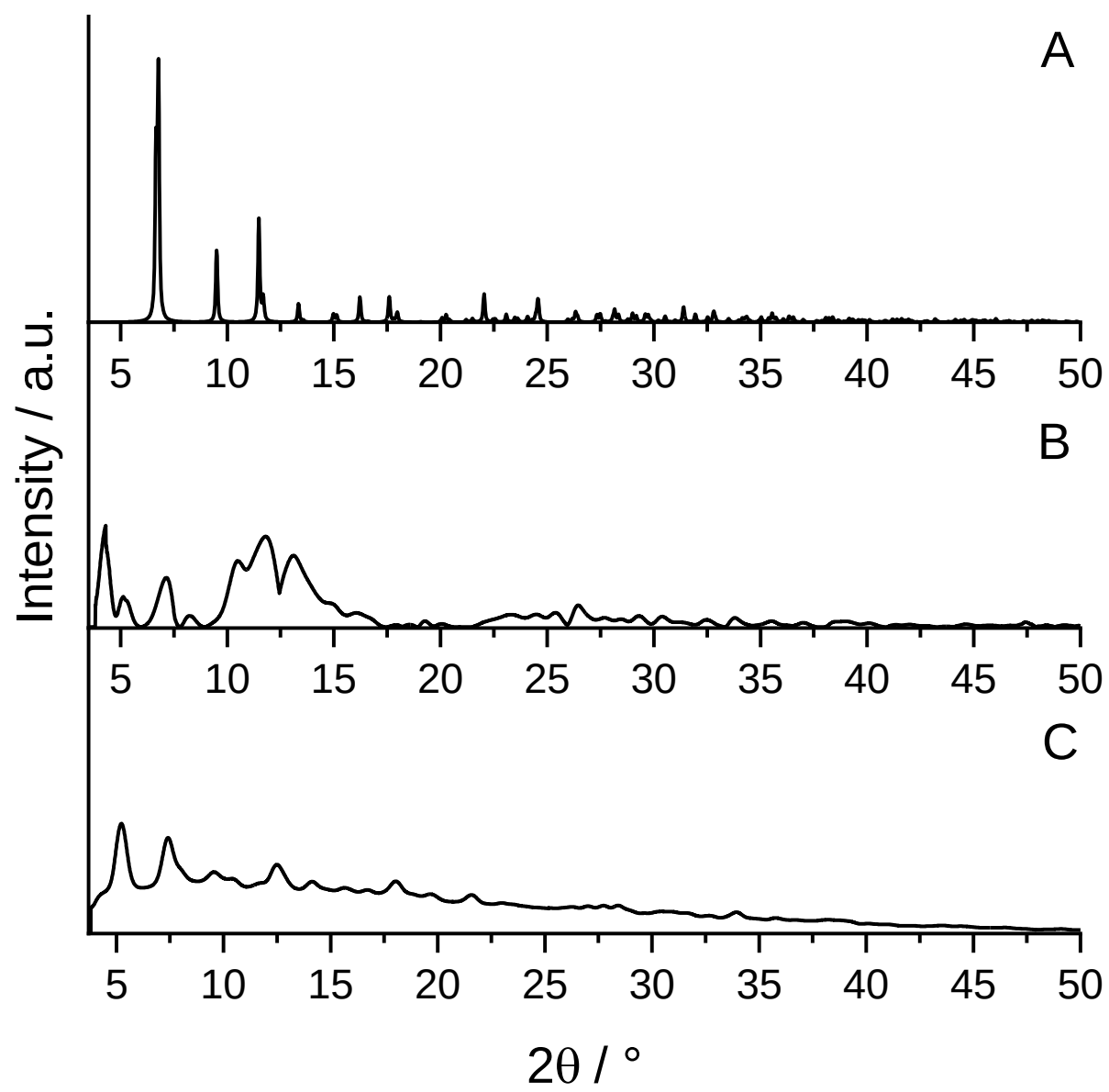

Figure S13. PXRD diffractograms of polymer **6**. **A**: Simulated PXRD pattern calculated from the single-crystal X-ray structure. **B**: Experimental PXRD pattern of single crystals of **6**. The measurement was performed immediately after removal of the crystals from the mother liquor. **C**: Experimental PXRD pattern of crystalline **6** after drying under vacuum. The data indicate rapid loss of crystallinity, even before drying under vacuum.

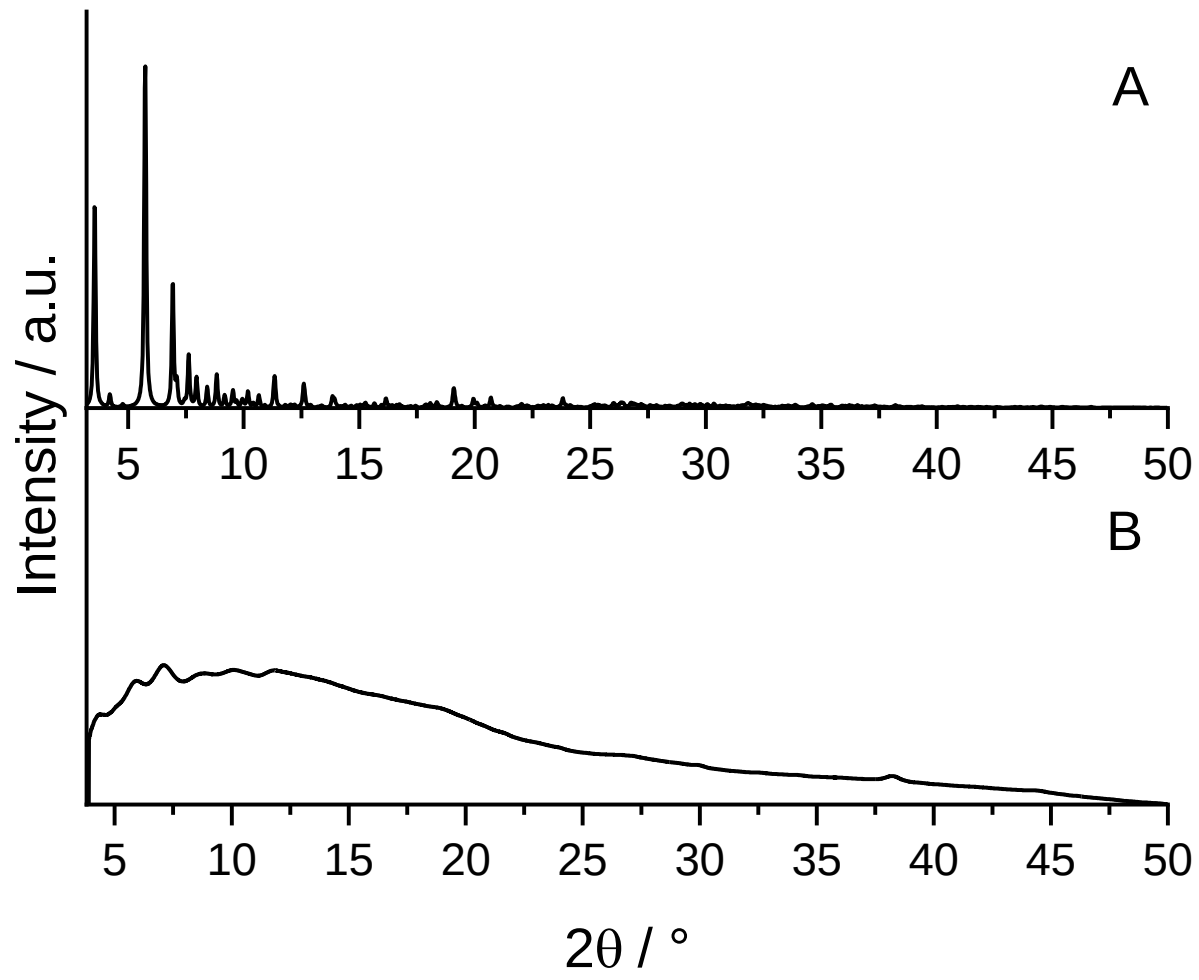

Figure S14. PXRD diffractograms of polymer **7**. **A**: Simulated PXRD pattern calculated from the single-crystal X-ray structure. **B**: Experimental PXRD pattern of single crystals of **7**. The measurement was performed immediately after removal of the crystals from the mother liquor. The data indicate rapid loss of crystallinity.

8. References

1. H. Kanazawa, E. R. T. Tiekink and K. Fujisawa, *Z. Kristallogr. - New Cryst. Struct.*, 2025, **240**, 443–445.

2. Q. Dong, T. Naren, L. Zhang, W. Jiang, M. Xue, X. Wang, L. Chen, C.-S. Lee, Q. Zhang, *Angew. Chem. Int. Ed.* 2024, **63**, e202405426.

3. G. M. Sheldrick, *Acta Crystallogr., Sect. A*, 2015, **71**, 3–8.

4. O. V. Dolomanov, L. J. Bourhis, R. J. Gildea, J. A. K. Howard and H. Puschmann, *J. Appl. Crystallogr.*, 2009, **42**, 339–341.

5. G. M. Sheldrick, *Acta Crystallogr., Sect. C*, 2015, **71**, 3–8.
